# Dual Regulation of Sprouty 4 Palmitoylation by ZDHHC7 and Palmitoyl-Protein Thioesterase 1: A Potential Therapeutic Strategy for Cisplatin-Resistant Osteosarcoma

**DOI:** 10.34133/research.0708

**Published:** 2025-05-23

**Authors:** Tianlong Huang, Yifan Chen, Qiangqiang Zhao, Xin Wu, Hongxing Li, Xin Luo, Yang Su, Shengqun Zhang, Pan Liu, Ning Tang

**Affiliations:** ^1^ Orthopaedic Department, The Second Xiangya Hospital of Central South University, Changsha, Hunan, China.; ^2^ Department of Hematology, Liuzhou People’s Hospital affiliated to Guangxi Medical University, Liuzhou, Guangxi, China.; ^3^ Department of Hematology, The Qinghai Provincial People’s Hospital, Xining, Qinghai, China.; ^4^ Department of Spine Surgery, Third Xiangya Hospital, Central South University, Changsha, Hunan, China.; ^5^ Department of Orthopaedic, The Central Hospital of Shaoyang, Shaoyang, China.; ^6^ Changsha Medical University, Changsha, Hunan, China.; ^7^ Orthopaedic Department, The Third Xiangya Hospital of Central South University, Changsha, Hunan, China.

## Abstract

**Background:** Osteosarcoma (OS) is a primary malignant bone tumor predominantly affecting adolescents. Chemotherapeutic agents, such as cisplatin, are commonly used in OS treatment; however, drug resistance markedly undermines treatment efficacy and contributes to reduced patient survival. The mechanisms underlying cisplatin resistance remain poorly understood. Recently, palmitoyl-protein thioesterase 1 (PPT1), a depalmitoylation enzyme, has attracted attention for its role in tumorigenesis and drug resistance. Investigating the mechanisms of PPT1 may offer new strategies to overcome resistance. **Methods:** This study analyzed multiple Gene Expression Omnibus datasets and utilized the OncoPredict tool to demonstrate the elevated expression of PPT1 in OS and its critical role in cisplatin resistance. By combining single-cell analysis with in vitro and in vivo experiments, we explored how PPT1 influences OS development through depalmitoylation and assessed the antitumor effects of the PPT1 inhibitor Ezurpimtrostat (GNS561), as well as its synergistic effects when combined with cisplatin. **Results:** We demonstrated that Sprouty 4 (SPRY4) undergoes a dynamic palmitoylation cycle regulated by zinc finger DHHC-type palmitoyl transferase 7 (ZDHHC7) and PPT1, which modulates mitogen-activated protein kinase (MAPK) signaling and subsequently affects tumor cell proliferation, migration, apoptosis, and drug resistance. Further validation confirmed the effectiveness of the PPT1 inhibitor GNS561 in overcoming cisplatin resistance. Notably, GNS561 exhibited a significant synergistic effect when used in combination with cisplatin, greatly enhancing the sensitivity of cisplatin-resistant cells. **Conclusion:** This study highlights the pivotal role of PPT1 in OS resistance mechanisms. PPT1 and ZDHHC7 regulate SPRY4 through a dynamic palmitoylation–depalmitoylation cycle that modulates MAPK signaling activation and contributes to OS cell proliferation, migration, and drug resistance. As a PPT1 inhibitor, GNS561 not only inhibits OS cell proliferation but also demonstrates synergistic effects with cisplatin, significantly enhancing cisplatin sensitivity in resistant cells and promoting apoptosis. Our findings offer a novel approach for targeting PPT1 in therapeutic strategies. GNS561 holds promise as an adjunctive therapy when combined with cisplatin, potentially overcoming resistance and improving efficacy, thereby enhancing the prognosis for OS patients. Future studies should further investigate the clinical potential of GNS561 and optimize OS treatment strategies.

## Introduction

Osteosarcoma (OS) is a primary malignant bone tumor, with the highest incidence in adolescents and individuals over 60 years of age [1]. The estimated annual incidence in children and adolescents has risen to between 3 and 4.5 cases per million, peaking during the pubertal growth spurt, especially at the rapidly growing metaphyseal ends of long bones [2]. Cisplatin, a widely used chemotherapeutic agent, exerts its cytotoxic effects by disrupting DNA replication [3]. In recent years, treatment advances have been made with surgery combined with the MAP regimen, which includes cisplatin, methotrexate, and doxorubicin [4,5]. However, chemotherapy resistance not only reduces treatment efficacy but also markedly lowers long-term survival rates, increasing the risk of recurrence and metastasis. For patients with metastatic or recurrent disease, the 5-year survival rate remains below 20% [6,7]. Resistance to chemotherapy has become a major therapeutic challenge, and postsurgical resistance to adjuvant chemotherapy further heightens the likelihood of recurrence and metastasis. The mechanisms underlying cisplatin resistance remain incompletely understood, with potential factors including reduced drug accumulation in tumor cells, inactivation of cisplatin following binding to intracellular glutathione and metallothioneins, and enhanced DNA repair by tumor cells to evade immune attack [8–10]. The combined effects of these mechanisms markedly impair the anticancer efficacy of cisplatin. Despite progress in understanding these mechanisms, no effective clinical strategies have been developed to overcome cisplatin resistance.

Recent studies have revealed that palmitoyl-protein thioesterase 1 (PPT1) is closely associated with the initiation, progression, and drug resistance of various malignant tumors [11–13]. PPT1 functions as a critical depalmitoylation enzyme, while palmitoylation, a key posttranslational modification, involves the addition of palmitoyl groups to proteins. This modification influences protein membrane localization, stability, and function, thereby regulating biological processes such as cell signaling, proliferation, and differentiation [14,15]. Depalmitoylation, the reverse of palmitoylation, is catalyzed by enzymes like PPT1, which markedly affect cellular behavior. In various tumor types, PPT1 exhibits consistent functions that are closely tied to tumor progression, immune evasion, and chemotherapy resistance. Studies have shown that PPT1 is overexpressed in multiple malignant tumors and is strongly linked to tumor progression. For example, in oral squamous cell carcinoma, PPT1 promotes tumor cell proliferation, migration, and invasion, thereby enhancing tumor cell survival [13,16]. In hepatocellular carcinoma, PPT1 induces resistance to sorafenib by regulating autophagy [12,17]. These findings suggest that PPT1 may influence chemotherapy efficacy by modulating the metabolism and survival mechanisms within tumor cells. Therefore, further investigation into the molecular mechanisms underlying OS drug resistance, along with the development of targeted therapeutic strategies, is essential to improve the prognosis of OS patients.

Building on the previous background, we hypothesize that PPT1 regulates the initiation and progression of OS through depalmitoylation mechanisms. Specifically, PPT1 may enhance drug resistance, as well as tumor proliferation and invasion, in OS cells via depalmitoylation. If validated, this hypothesis will elucidate the specific mechanisms of PPT1 in OS and identify potential new therapeutic targets. Therefore, investigating the role of PPT1 in OS not only is scientifically significant but also holds promise for providing novel therapeutic strategies to improve the prognosis of OS patients.

## Results

### Elevated expression of PPT1 in OS

**Fig. 1.**
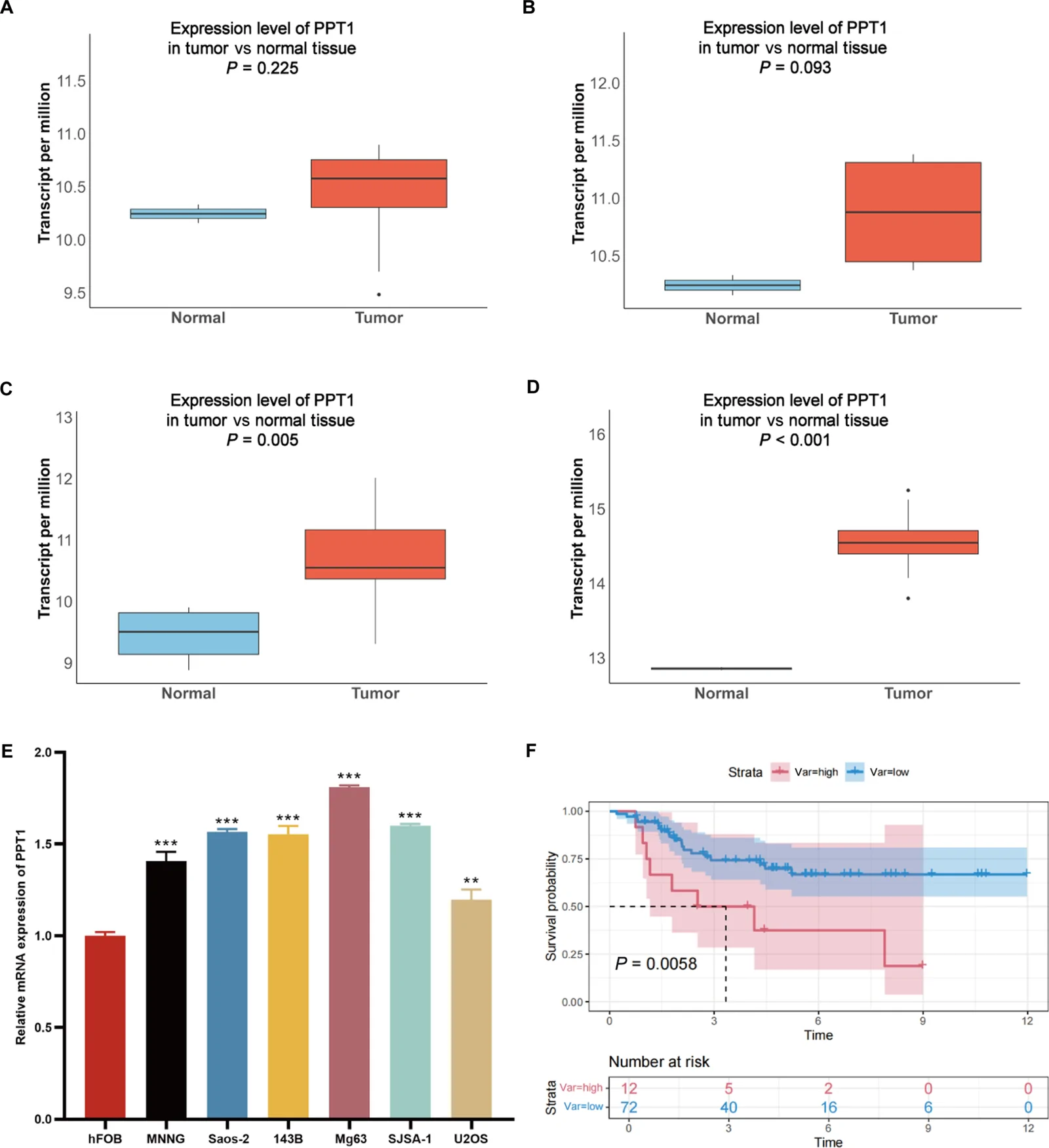
PPT1 overexpression in osteosarcoma (OS). (A to D) Expression levels of PPT1 in OS tissues compared to normal tissues, analyzed using datasets GSE12865 (A), GSE11416 (B), GSE28425 (C), and GSE14359 (D). (E) Relative mRNA expression levels of PPT1 in the normal osteoblast cell line hFOB (*n* = 3) and OS cell lines MNNG, Saos-2, 143B, Mg63, SJSA-1, and U2OS (*n* = 3 for each group). ns, not significant; *P < 0.05; **P < 0.01; ***P < 0.001 compared with hFOB normal cells. (F) Kaplan–Meier survival curve for PPT1 expression in OS patients from the TARGET dataset.

To explore the potential role of PPT1 in OS, we conducted a comprehensive analysis of several publicly available datasets, including GSE11416, GSE12865, GSE28425, and GSE14359, using bioinformatics approaches. The results revealed that PPT1 expression was significantly elevated in primary OS tumor tissues compared to normal tissues (Fig. 1A to D). This finding was further corroborated by analysis of PPT1 expression in different cell lines: compared to normal osteoblast cell lines, PPT1 was markedly up-regulated in various OS cell lines, with the highest expression in MG63 cells and the lowest among OS cells in U2OS cells (Fig. 1E). To assess the relationship between PPT1 expression and patient prognosis, Kaplan–Meier survival analyses were performed using the TARGET and GSE21257 databases. The results indicated that high PPT1 expression was significantly associated with poor prognosis in OS patients (Fig. 1F and Fig. S1). These findings suggest that PPT1 plays a pivotal role in the onset and progression of OS. Therefore, we conclude that the elevated expression of PPT1 in OS tissues and cells is closely linked to poor prognosis and may markedly contribute to OS progression.

### Role of elevated PPT1 expression in multidrug resistance in OS

**Fig. 2.**
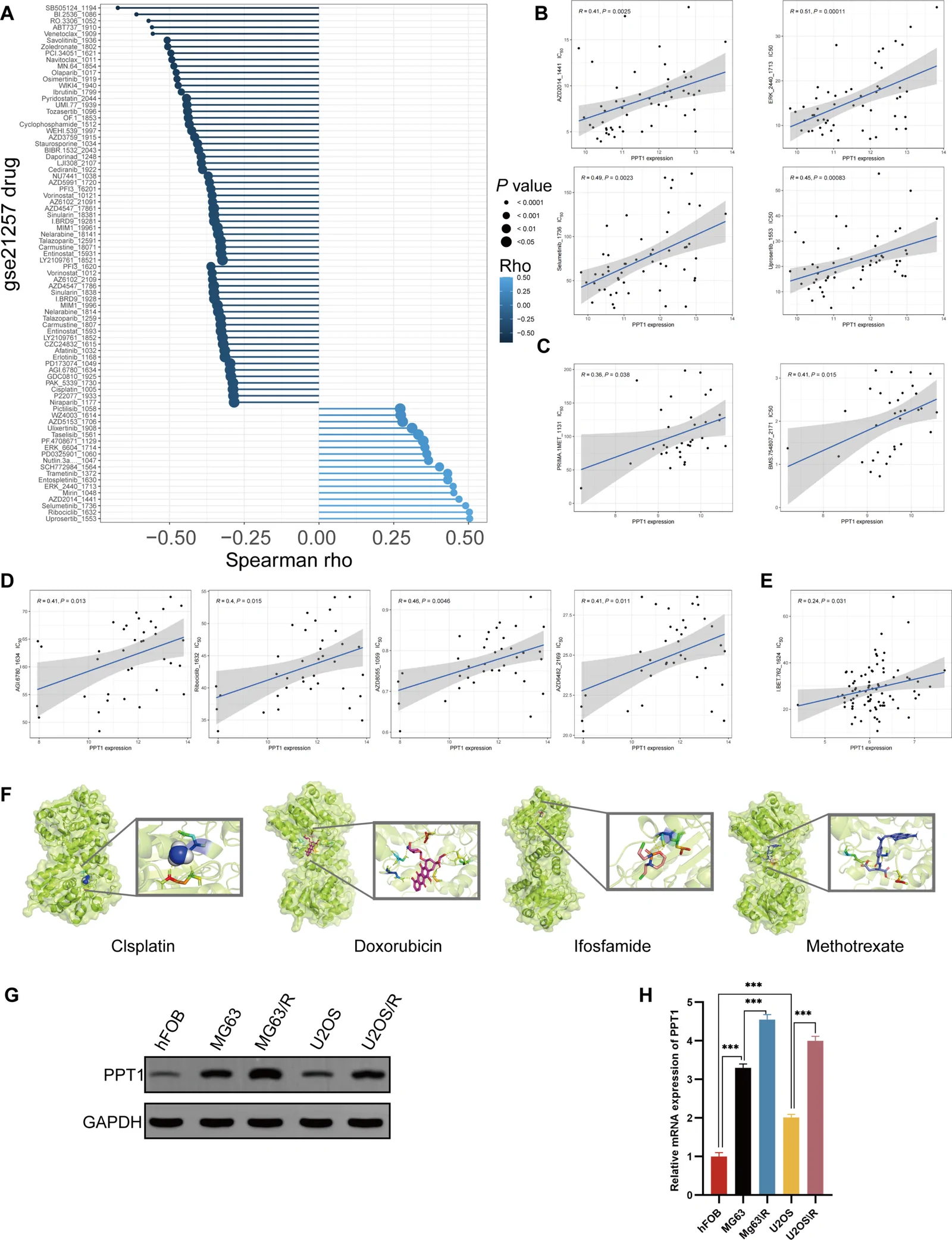
Elevated PPT1 expression and its role in multidrug resistance in OS. (A) Spearman correlation coefficients depicting the relationship between PPT1 gene expression and the sensitivity of 198 drugs in the GSE21257 dataset. (B) Scatter plot with linear regression analysis illustrating the association between PPT1 gene expression and drug resistance in the GSE21257 dataset. (C to E) Scatter plots and linear regression analyses of PPT1 gene expression and drug sensitivity correlations in the GSE16091 (C), GSE39055 (D), and TARGET (E) datasets. (F) Molecular docking results showing interactions between the PPT1 protein and 4 anticancer drugs: cisplatin, doxorubicin, ifosfamide, and methotrexate. The left panels present the overall structures of the docking complexes, while the right panels provide magnified views of the docking sites, highlighting the interaction details between drug molecules and the PPT1 protein. The PPT1 protein is represented in green, and drug molecules are depicted in different colors. (G) Western blot analysis of PPT1 protein expression levels across various cell lines. (H) qRT-PCR analysis comparing PPT1 gene expression levels in OS cells and their cisplatin-resistant counterparts. ***P < 0.001.

The prognosis of OS patients is often markedly impacted by chemotherapy resistance. To investigate the potential role of PPT1 in drug sensitivity, we utilized the OncoPredict algorithm to systematically analyze gene expression data from multiple datasets, including GSE16091, GSE39055, TARGET, and GSE21257. The analysis revealed that elevated PPT1 expression was strongly correlated with increased 50% inhibitory concentration (IC_50_ )values for various drugs, suggesting a pivotal role for PPT1 in multidrug resistance mechanisms (Fig. 2A to E). To further support this hypothesis, molecular docking analyses were conducted to examine the direct interactions between the PPT1 protein and 4 commonly used first-line chemotherapy drugs for OS: cisplatin, doxorubicin, ifosfamide, and methotrexate. The results identified multiple potential binding sites between PPT1 and these drug molecules, providing additional evidence for PPT1’s involvement in OS resistance mechanisms (Fig. 2F). Moreover, cisplatin-resistant OS cell lines were established [18,19], and elevated PPT1 expression levels were confirmed using reverse transcription quantitative polymerase chain reaction (RT-qPCR) and Western blot analyses (Fig. 2G and H). These findings indicate that PPT1 overexpression not only is associated with chemotherapy resistance in OS cells but also may drive resistance development through direct interactions with chemotherapeutic agents. In conclusion, our study underscores the critical role of PPT1 in cisplatin resistance in OS and identifies it as a promising therapeutic target for overcoming drug resistance in future treatments.

### PPT1 enhances proliferation, migration, and invasion while suppressing apoptosis in cisplatin-resistant OS cell lines

**Fig. 3.**
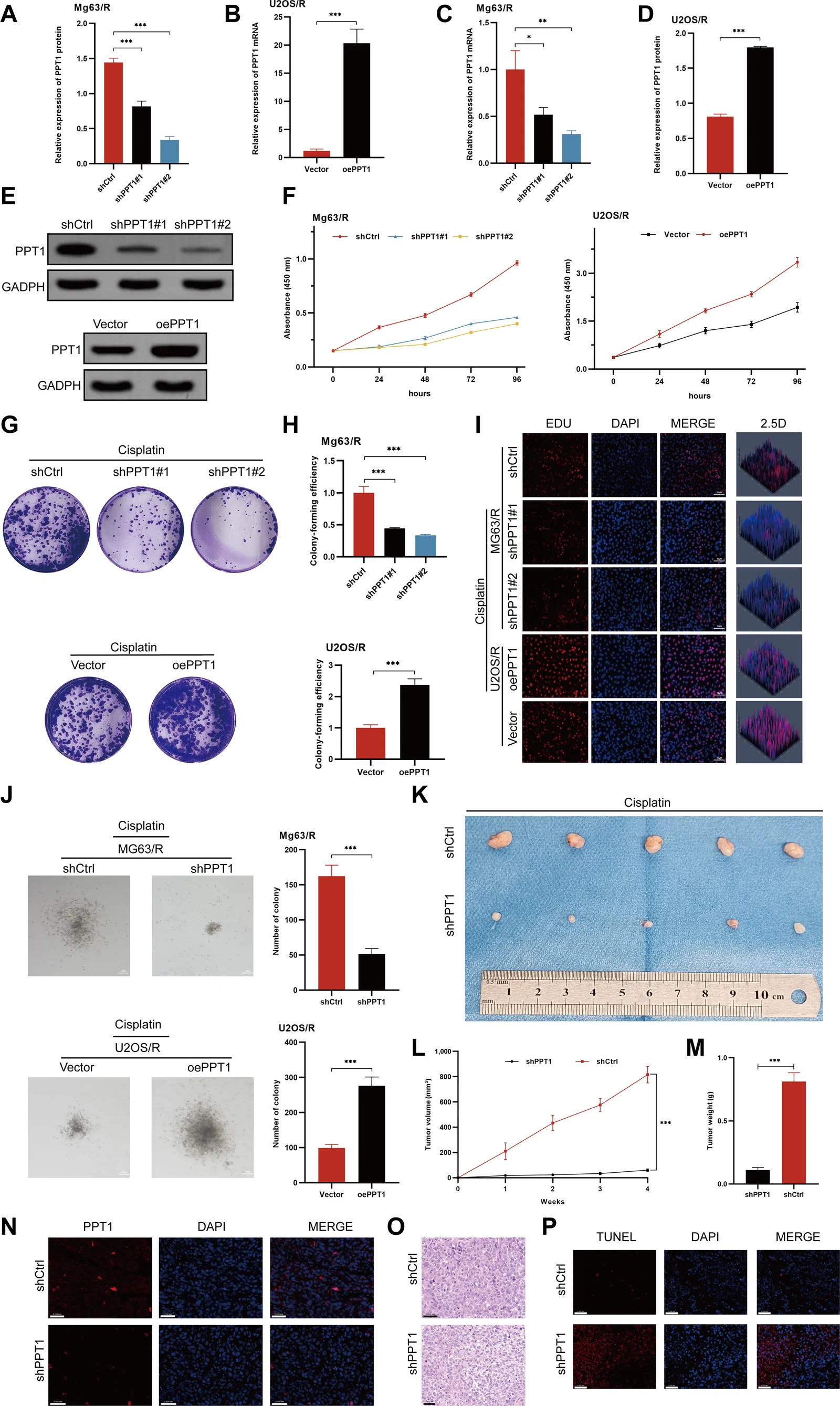
The effects of PPT1 on OS cell proliferation in vitro. (A to D) Quantitative analysis of mRNA and protein expression levels in MG63/R and U2OS/R cells following PPT1 knockdown or overexpression. (E) Western blot analysis of PPT1 protein levels in cisplatin-treated MG63/R and U2OS/R cells after PPT1 knockdown or overexpression. (F) CCK-8 assay assessing the effects of PPT1 knockdown or overexpression on cell proliferation. (G) Representative images of colony formation assays in MG63/R and U2OS/R cells following PPT1 knockdown or overexpression (*n* = 3). (H) Statistical analysis of colony formation capacity in MG63/R and U2OS/R cells after PPT1 knockdown or overexpression. (I) EdU incorporation assay evaluating proliferation in MG63/R and U2OS/R cells following PPT1 knockdown or overexpression (*n* = 3). Scale bar, 50 μm. (J) Soft agar colony formation assay illustrating anchorage-independent growth of MG63/R and U2OS/R cells after PPT1 knockdown or overexpression (*n* = 3). Scale bar, 50 μm. The left panel displays representative images, and the right panel provides statistical analysis. (K) Representative tumor images from in vivo experiments showing the effects of PPT1 knockdown or overexpression on tumor growth in xenograft models. (L) Tumor volume growth curves in different mouse groups, calculated as (length × width^2^)/2. (M) Comparison of tumor weights among the different mouse groups. (N) Immunofluorescence analysis of PPT1 expression levels in tumor tissues (*n* = 3). Scale bar, 50 μm. (O) Hematoxylin and eosin (H&E) staining for histological analysis of tumor tissues (*n* = 3). Scale bar, 50 μm. (P) TUNEL assay quantifying apoptosis levels in tumor tissues from PPT1 knockdown and control groups (*n* = 3). Scale bar, 50 μm.*P < 0.05, **P < 0.01, ***P < 0.001.

To explore the biological function of PPT1 in the progression of OS, we established PPT1 knockdown and overexpression cell models in cisplatin-resistant OS cell lines. qRT-PCR and Western blot analyses confirmed significant alterations in PPT1 expression levels across treatment groups, demonstrating successful model construction (Fig. 3A to E). In vitro functional assays showed that under cisplatin treatment, PPT1 knockdown significantly inhibited cell proliferation, whereas PPT1 overexpression markedly enhanced it. EdU incorporation, colony formation, and soft agar assays consistently indicated that PPT1 knockdown significantly reduced cell proliferation and clonogenic capacity, while PPT1 overexpression increased these abilities (Fig. 3F to J).

In vivo experiments further revealed that PPT1 expression levels in OS cells significantly affected tumor growth. Tumors derived from PPT1-knockdown cells exhibited substantially reduced volume and weight compared to the control group, whereas tumors in the PPT1-overexpression group displayed notable increases in both parameters (Fig. 3K to M). Immunofluorescence analysis demonstrated a significant increase in apoptosis within tumor tissues from the PPT1-knockdown group. Furthermore, hematoxylin and eosin (H&E) staining and terminal deoxynucleotidyl transferase dUTP nick end labeling (TUNEL) assays confirmed that PPT1 inhibits tumor cell apoptosis (Fig. 3N to P). Collectively, these findings highlight the critical role of PPT1 in OS progression by promoting cell proliferation, migration, and invasion, while suppressing apoptosis.

To further validate the functional role of PPT1 in OS cell migration and invasion, Transwell migration and invasion assays were conducted. The results revealed that PPT1 knockdown significantly suppressed the migration and invasion abilities of MG63/R cells, whereas PPT1 overexpression markedly enhanced these capabilities in U2OS/R cells (Fig. S2A and B). These findings indicate that PPT1 may drive the invasive progression of OS by facilitating migration and invasion.

To evaluate the impact of PPT1 on the cell cycle, flow cytometry was used to analyze the effects of PPT1 knockdown and overexpression. PPT1 knockdown led to a significant increase in the proportion of cells in the G1/G0 phase and a concomitant decrease in the S and G2/M phases, suggesting that PPT1 deficiency may induce cell cycle arrest (Fig. S2C). Conversely, PPT1 overexpression facilitated cell entry into the S and G2/M phases, indicating that PPT1 up-regulation may promote cell cycle progression and enhance proliferative capacity.

Furthermore, to investigate the role of PPT1 in apoptosis, apoptosis levels were assessed using flow cytometry. PPT1 knockdown significantly increased the proportions of early and late apoptotic cells, whereas PPT1 overexpression markedly reduced apoptosis rates (Fig. S2D and E). These results highlight a potential role of PPT1 in suppressing apoptosis.

To corroborate these findings, Western blot analysis was performed to assess the expression of apoptosis- and epithelial–mesenchymal transition (EMT)-related proteins. PPT1 knockdown up-regulated the expression of pro-apoptotic proteins BAX and cleaved caspase-3 while down-regulating the anti-apoptotic protein BCL2, further confirming the role of PPT1 in promoting apoptosis (Fig. S2F). Moreover, PPT1 knockdown significantly inhibited the EMT process, as demonstrated by increased E-cadherin expression and decreased N-cadherin and vimentin expression (Fig. S2G). These findings suggest that PPT1 may enhance the migratory and invasive capacities of OS cells by driving EMT.

Furthermore, we examined whether PPT1 overexpression could enhance cisplatin resistance in the parental OS cell lines MG63 and U2OS. As shown in Fig. S3A, PPT1 was overexpressed in these cell lines, after which their sensitivity to cisplatin was assessed. Following cisplatin treatment, the PPT1-overexpressing group exhibited significantly higher IC_50_ values in the Cell Counting Kit-8 (CCK-8) assay compared to the control group, indicating that PPT1 overexpression increased cisplatin resistance in MG63 and U2OS cells (Fig. S3). Additionally, PPT1 overexpression markedly promoted cell proliferation, migration, and invasion, while significantly reducing apoptosis (Fig. S3C to H). These results further support the role of PPT1 in mediating chemoresistance in tumor cells.

In summary, PPT1 plays a pivotal role in the malignant progression of OS by promoting cell proliferation, migration, and invasion; suppressing apoptosis; and enhancing cisplatin resistance.

To investigate the effects of PPT1 knockdown on trabecular and cortical bone in an orthotopic OS model, we performed 3-dimensional reconstruction and bone structure analysis of femurs and tibias from the shPPT1 and control (shNC) groups using micro-CT technology, followed by quantification of relevant bone parameters.

**Fig. 4.**
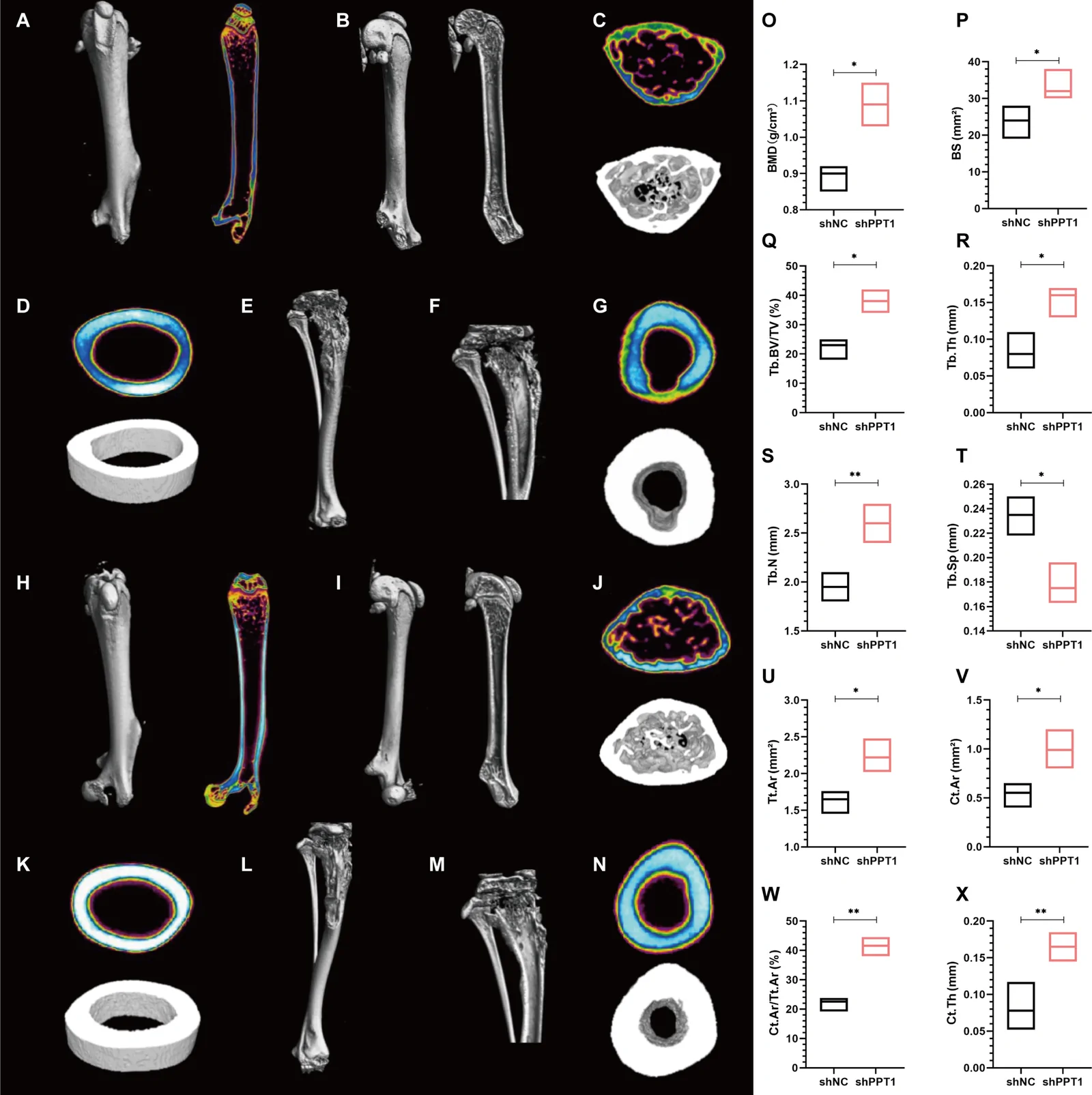
Impact of PPT1 knockdown on bone structure in an orthotopic OS model. (A to D) Three-dimensional reconstruction and coronal view of the femur in the control group (A); sagittal CT image (B); pseudocolor cross-sectional density map and transverse 3-dimensional reconstruction at the proximal femur (C); pseudocolor cross-sectional density map and 3-dimensional reconstruction of cortical bone at the femoral midshaft (D). (E to G) Three-dimensional reconstruction of the tibia in the control group (E); coronal 3-dimensional reconstruction of the tibia (F); pseudocolor cross-sectional density map and 3-dimensional reconstruction of cortical bone at the tibial midshaft (G). (H to K) Three-dimensional reconstruction and coronal view of the femur in the shPPT1 group (H); sagittal CT image (I); pseudocolor cross-sectional density map and transverse structure at the proximal femur (J); pseudocolor cross-sectional density map and 3-dimensional reconstruction of cortical bone at the femoral midshaft (K). (L to N) Three-dimensional reconstruction of the tibia in the shPPT1 group (L); coronal CT image (M); pseudocolor cross-sectional density map and transverse structure of cortical bone at the tibial midshaft (N). (O to X) Bar graphs comparing femoral bone parameters between groups, including bone mineral density (BMD, O); bone surface area (BS, P); trabecular bone volume fraction (Tb.BV/TV, Q); trabecular thickness (Tb.Th, R); trabecular number (Tb.N, S); trabecular spacing (Tb.Sp, T); total cross-sectional area (Tt.Ar, U); cortical area (Ct.Ar, V); cortical area fraction (Ct.Ar/Tt.Ar, W); and cortical thickness (Ct.Th, X). Statistical significance: *P < 0.05, **P < 0.01.

Three-dimensional reconstruction and cross-sectional CT images revealed significant differences between the groups (Fig. 4A to N). In the shPPT1 group, femurs (Fig. 4H) and tibias (Fig. 4L) exhibited increased bone density and more compact trabeculae. Conversely, in the control group, femurs (Fig. 4A) and tibias (Fig. 4E) showed reduced bone density and loose trabecular structures, with the tibias displaying more severe damage.

Pseudocolor cross-sectional density maps further confirmed these observations. In the femurs (Fig. 4J), the shPPT1 group demonstrated significantly denser and more abundant trabeculae, reduced bone resorption, and higher bone density. In contrast, the femurs in the control group (Fig. 4C) showed sparse trabeculae, exacerbated bone resorption, and lower bone density. Similarly, in the tibias (Fig. 4N), the shPPT1 group displayed a greater proportion of high-density areas (cool colors), indicating enhanced bone density and reduced bone destruction. In contrast, the control group’s tibias (Fig. 4G) predominantly exhibited low-density areas (warm colors), reflecting osteoporosis and severe bone damage. These findings suggest that PPT1 knockdown mitigates bone loss and improves bone density, with distinct pathological characteristics observed in the femurs and tibias.

Quantitative analysis of bone parameters further validated these results (Fig. 4O to X). In the femurs, the shPPT1 group showed significant increases in bone mineral density (BMD, Fig. 4O), bone surface area (BS, Fig. 4P), trabecular bone volume fraction (Tb.BV/TV, Fig. 4Q), trabecular thickness (Tb.Th, Fig. 4R), trabecular number (Tb.N, Fig. 4S), cortical area (Ct.Ar, Fig. 4V), cortical area fraction (Ct.Ar/Tt.Ar, Fig. 4W), and cortical thickness (Ct.Th, Fig. 4X), along with a significant reduction in trabecular spacing (Tb.Sp, Fig. 4T). These findings indicate a denser and more robust trabecular structure in the shPPT1 group.

In conclusion, PPT1 knockdown significantly enhances bone structure, increases bone density, and reduces trabecular and cortical bone destruction in the OS model. These findings suggest that PPT1 may play a critical role in promoting bone loss and structural damage.

### Comprehensive analysis of PPT1 using single-cell RNA sequencing data

To elucidate the potential mechanisms of the PPT1 gene in OS, we integrated and systematically analyzed 2 single-cell RNA sequencing (scRNA-seq) datasets (GSE152048 and GSE162454). First, we identified highly variable genes, with red dots in Fig. S4A representing the 3,000 selected highly variable genes and black dots indicating nonvariable genes (23,578 in total). Identifying highly variable genes enables effective cell clustering and functional analysis. Next, principal component analysis (PCA) was conducted to evaluate differences between cell cycle phases and sample groups. Figure S4B highlights significant gene expression differences across cell cycle phases, while Fig. S4C illustrates batch effects between sample groups, necessitating batch correction for further analyses.

During quality control (QC), we visualized RNA counts and other QC metrics. Figure S4D shows the distribution of nCount_RNA (RNA molecules detected per cell), percent.mt (mitochondrial gene proportion), nFeature_RNA (number of genes detected per cell), and percent.HB (hemoglobin gene proportion) across sample groups. Figure S4E presents violin plots of these QC metrics, assessing their distribution differences among sample groups. Following QC, 151,656 high-quality cells were selected for subsequent single-cell analysis.

We used a Clustree plot (Fig. S4F) to visualize cell clustering results at different resolutions, selecting the optimal resolution to ensure accurate clustering. After batch correction, t-distributed stochastic neighbor embedding (t-SNE) and uniform manifold approximation and projection (UMAP) plots confirmed uniform cell distribution, indicating successful elimination of batch effects (Fig. S4G). Prominent cell clusters were identified (Fig. S4H and I). Figure S4J depicts the proportions of various cell types in each sample group, with distinct colors representing different cell types, facilitating the identification of intergroup differences in cell-type composition.

To further investigate the role of the PPT1 gene in OS, we isolated malignant osteoblasts and conducted an integrated analysis of scRNA-seq data.

**Fig. 5.**
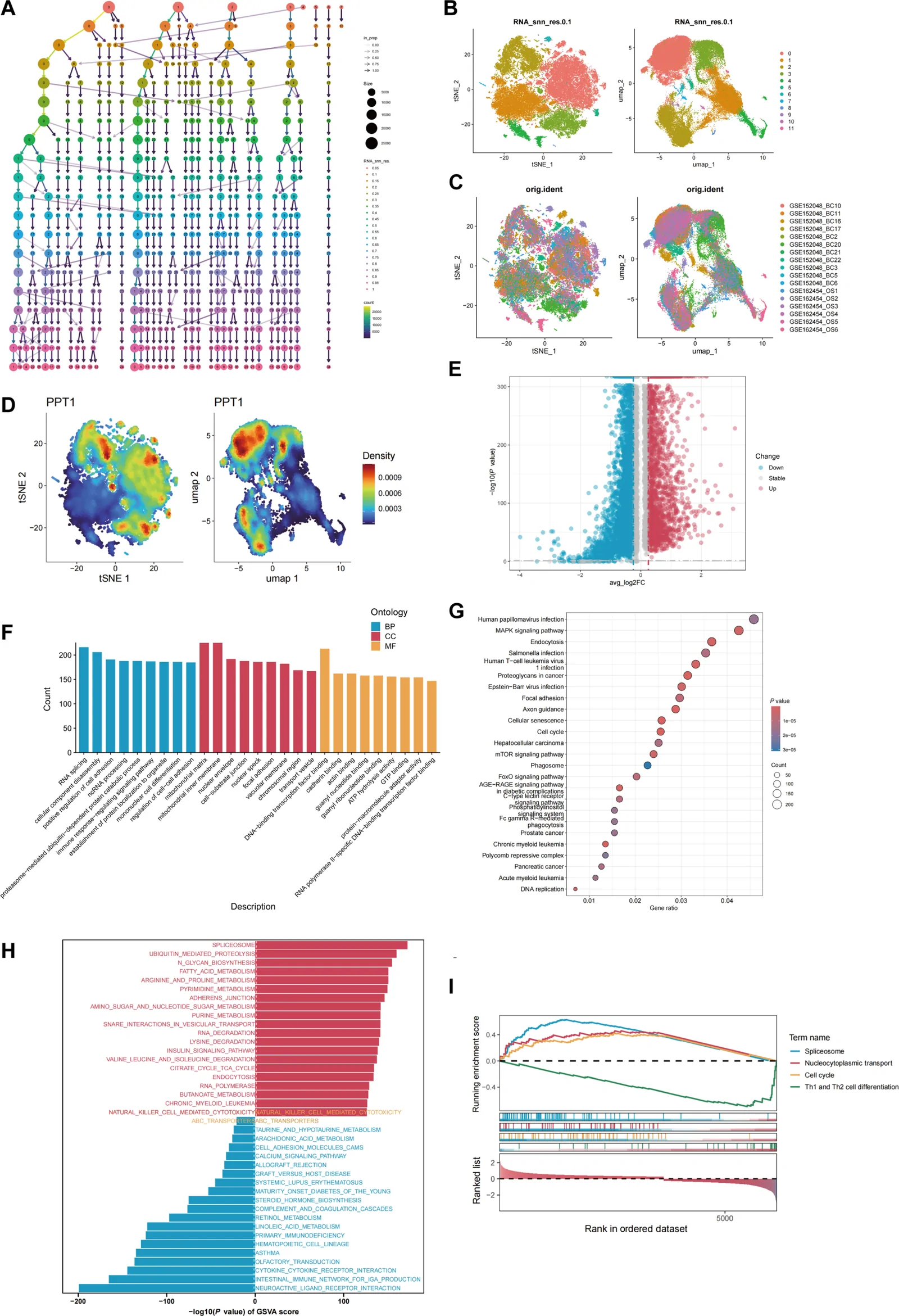
Integrated analysis of PPT1 in single-cell RNA sequencing data. (A) Clustree plot displaying clustering structures across varying resolutions, highlighting the distribution patterns of PPT1 expression among subpopulations. (B and C) t-SNE and UMAP plots illustrating cell clustering and intersample differences. (D) PPT1 expression density maps in t-SNE and UMAP spaces, with a gradient from blue (low density) to red (high density). (E) Volcano plot showing differential expression analysis of PPT1, where red indicates genes up-regulated in the PPT1 high-expression group and blue indicates those down-regulated. (F) GO enrichment analysis presented as bar charts, highlighting significant biological processes (BP), cellular components (CC), and molecular functions (MF) associated with PPT1. (G) KEGG pathway enrichment analysis shown as a bubble chart, where bubble size represents gene counts and color reflects *P* values. (H) Gene set variation analysis (GSVA) bar chart depicting differences in functional gene set activities between high and low PPT1 expression groups. (I) Gene set enrichment analysis (GSEA) results with curves indicating gene set enrichment profiles within ranked datasets. The *x*-axis represents gene ranks, and the *y*-axis denotes enrichment scores.

Using a Clustree plot (Fig. 5A), we determined the optimal resolution for clustering malignant osteoblasts to be 0.1. At this resolution, t-SNE and UMAP dimensionality reduction plots (Fig. 5B and C) revealed 11 distinct clusters, enabling the identification of subpopulations and providing an initial overview of heterogeneity within malignant osteoblasts. To evaluate PPT1 expression, we generated t-SNE and UMAP density plots (Fig. 5D), which showed high PPT1 expression in malignant osteoblasts. Based on PPT1 expression levels, cells were divided into high- and low-expression groups using the median expression value. Differential expression analysis between these groups was visualized in a volcano plot (Fig. 5E), highlighting significantly altered genes.

To elucidate the biological functions and mechanisms of PPT1, we performed Gene Ontology (GO) enrichment analysis, Kyoto Encyclopedia of Genes and Genomes (KEGG) pathway enrichment analysis, Gene Set Variation Analysis (GSVA), and Gene Set Enrichment Analysis (GSEA) on the differentially expressed genes.

GO analysis (Fig. 5F) revealed that PPT1 is enriched in biological processes such as protein regulatory signaling pathways, immune responses, and cell adhesion, indicating its key roles in signal transduction, immune regulation, and intercellular interactions. In terms of cellular components, PPT1 was linked to the extracellular matrix, nuclear matrix, and chromatin regions, suggesting involvement in cytoskeletal reorganization and gene expression regulation. At the molecular function level, PPT1 was associated with adenosine triphosphate (ATP) binding, nucleotide binding, and catalytic activity, underscoring its roles in energy metabolism and signal transduction. KEGG analysis showed significant enrichment of differentially expressed genes in cancer-related and drug resistance pathways, including the mitogen-activated protein kinase (MAPK), mammalian target of rapamycin (mTOR), and FoxO signaling pathways (Fig. 5G). These results emphasize PPT1’s influence on tumor cell behavior, particularly in drug resistance, tumorigenesis, and signal transduction. The significant enrichment of the MAPK signaling pathway highlights its role in promoting tumor cell proliferation and survival. GSVA (Fig. 5H) and GSEA (Fig. 5I) revealed up-regulation of functional and metabolic pathways in cells with high PPT1 expression, especially those related to drug resistance and tumor progression. Notably enriched pathways included the spliceosome, nucleocytoplasmic transport, ubiquitin-mediated protein degradation, and pathways associated with cell cycle and division. These findings suggest that PPT1 contributes to OS progression by engaging in these malignant pathways and provide insights into its role in tumorigenesis and therapeutic targeting.

In summary, integrating scRNA-seq data from all cells and malignant osteoblasts revealed the expression characteristics and potential functions of the PPT1 gene in OS. Screening for highly variable genes, QC analyses, and clustering provided a comprehensive perspective on cellular heterogeneity. Further analysis of malignant osteoblasts identified significant differences in PPT1 expression across cell populations. Differential expression and functional enrichment analyses highlighted PPT1-related genes’ involvement in key biological processes and signaling pathways, particularly the MAPK signaling pathway, underscoring PPT1’s pivotal role in OS progression. These results are consistent with our previous cellular experiments, further confirming PPT1’s critical role in OS development.

### PPT1 and ZDHHC7 antagonistically regulate SPRY4 palmitoylation and MAPK activation

**Fig. 6.**
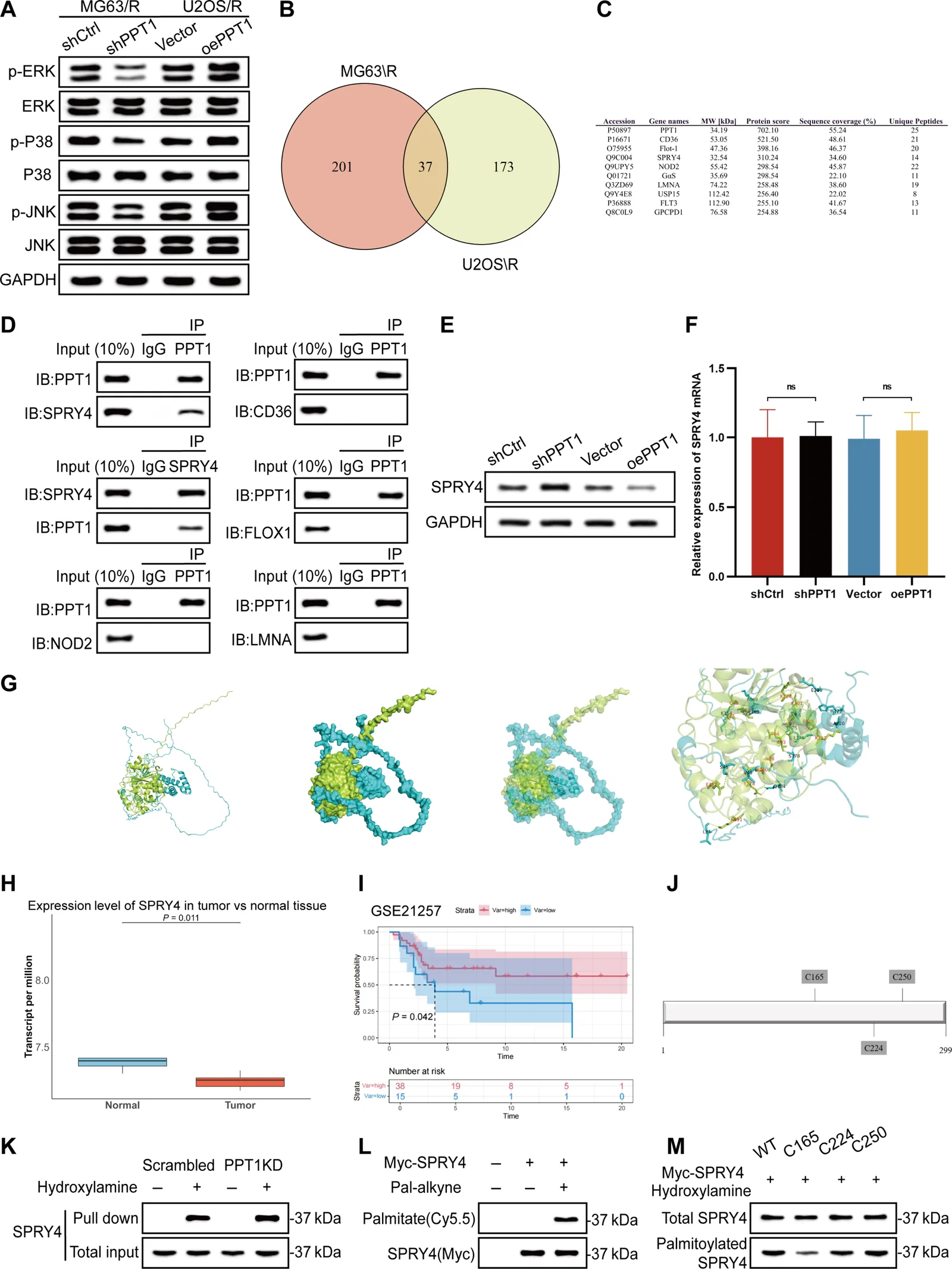
PPT1 modulates SPRY4 palmitoylation to regulate the MAPK signaling pathway in OS cells. (A) Western blot analysis of phosphorylated MAPK signaling proteins (p-ERK, p-p38, and p-JNK) in MG63/R and U2OS/R cells after PPT1 knockdown, demonstrating PPT1’s regulatory role in MAPK signaling. (B) Venn diagram of PPT1-interacting proteins identified through liquid chromatography–tandem mass spectrometry (LC-MS/MS) in MG63/R and U2OS/R cells, with overlapping regions indicating common interacting proteins. (C) List of potential PPT1-interacting proteins identified by mass spectrometry in MG63/R and U2OS/R cell lines. (D) Co-immunoprecipitation experiments revealing the direct interaction between PPT1 and SPRY4 and co-precipitation results with CD36, FLOX1, NOD2, and LMNA. (E) Western blot analysis of SPRY4 protein expression levels following PPT1 knockdown and overexpression. (F) Relative mRNA expression levels of SPRY4 after PPT1 knockdown and overexpression. (G) Molecular docking simulation predicting potential binding sites between PPT1 and SPRY4, providing insights into possible interaction mechanisms. (H) Analysis of SPRY4 expression levels in OS and normal tissues using GSE datasets. (I) Kaplan–Meier survival curve analysis showing a significant association between high SPRY4 expression and improved prognosis in OS patients. (J) Prediction of SPRY4 palmitoylation sites (C165, C224, and C250) using the Css-Palm software. (K) Resin-assisted capture (RAC) experiments demonstrating a significant increase in SPRY4 palmitoylation levels in MG63/R cells following PPT1 knockdown. (L) Click chemistry experiments further validating the increase in SPRY4 palmitoylation levels in MG63/R cells after PPT1 knockdown. (M) Western blot analysis showing a substantial reduction in SPRY4 palmitoylation in the C165 mutant, identifying C165 as a critical palmitoylation site. ns, not significant.

To investigate the mechanistic role of PPT1 in OS cells, we identified PPT1-interacting proteins and validated their regulatory roles in signaling pathways. Western blot analysis revealed that PPT1 knockdown significantly reduced the expression levels of key phosphorylated molecules in the MAPK signaling pathway, including p-ERK, p-p38, and p-JNK. These findings suggest that PPT1 may facilitate OS cell proliferation and drug resistance by activating the MAPK pathway (Fig. 6A).

To elucidate the molecular mechanisms underlying PPT1 function, we combined co-immunoprecipitation (Co-IP) with mass spectrometry to identify potential PPT1-interacting proteins (Fig. 6B and C). Across the MG63/R and U2OS/R cell lines, we identified 37 proteins interacting with PPT1, indicating their potential roles in PPT1-regulated biological processes. To pinpoint key interacting proteins, we selected the 5 most abundant candidates from the mass spectrometry results and validated their interactions using Co-IP experiments. The results demonstrated that PPT1 directly interacts with Sprouty 4 (SPRY4), whereas interactions with CD36, FLOX1, NOD2, and LMNA were negligible (Fig. 6D). As an inhibitor of the MAPK pathway, SPRY4 negatively regulates ERK activation via feedback inhibition. PPT1’s interaction with SPRY4 likely relieves this inhibition, thereby enhancing MAPK pathway activation. Additionally, analysis of the GSE28425 dataset revealed significantly lower SPRY4 expression in OS tissues compared to normal tissues (Fig. 6H). Kaplan–Meier survival analysis further showed that higher SPRY4 expression correlates with better prognosis in OS patients (Fig. 6I), suggesting that SPRY4 may function as a tumor suppressor in OS. Interestingly, our findings indicated that PPT1 knockdown significantly increased SPRY4 protein levels, while PPT1 overexpression decreased them (Fig. 6E). However, SPRY4 mRNA levels remained unchanged under these conditions (Fig. 6F), suggesting that PPT1 regulates SPRY4 protein stability and degradation via posttranslational modifications, such as depalmitoylation. Molecular docking analysis further supported this hypothesis, identifying multiple potential binding sites between PPT1 and SPRY4 (Fig. 6G).

Given PPT1’s depalmitoylating function, we hypothesized that PPT1 activates the MAPK pathway by regulating SPRY4 activity through depalmitoylation. Using Css-Palm software, we identified C165, C224, and C250 as potential SPRY4 palmitoylation sites (Fig. 6J). In PPT1-knockdown MG63/R cells, resin-assisted capture (RAC) experiments revealed a significant increase in SPRY4 palmitoylation levels (Fig. 6K). Click chemistry experiments corroborated this finding, demonstrating that PPT1 knockdown significantly enhanced SPRY4 palmitoylation (Fig. 6L). To identify the critical palmitoylation site, we generated SPRY4 mutants at C165, C224, and C250. Mutations at C165 significantly reduced SPRY4 palmitoylation, confirming C165 as the key palmitoylation site (Fig. 6M).

In conclusion, our study demonstrates that PPT1 activates the MAPK signaling pathway by regulating SPRY4 depalmitoylation, thereby promoting OS cell growth and cisplatin resistance. This discovery underscores the potential role of PPT1 as a regulatory factor in OS development and progression and highlights its impact on patient prognosis.

**Fig. 7.**
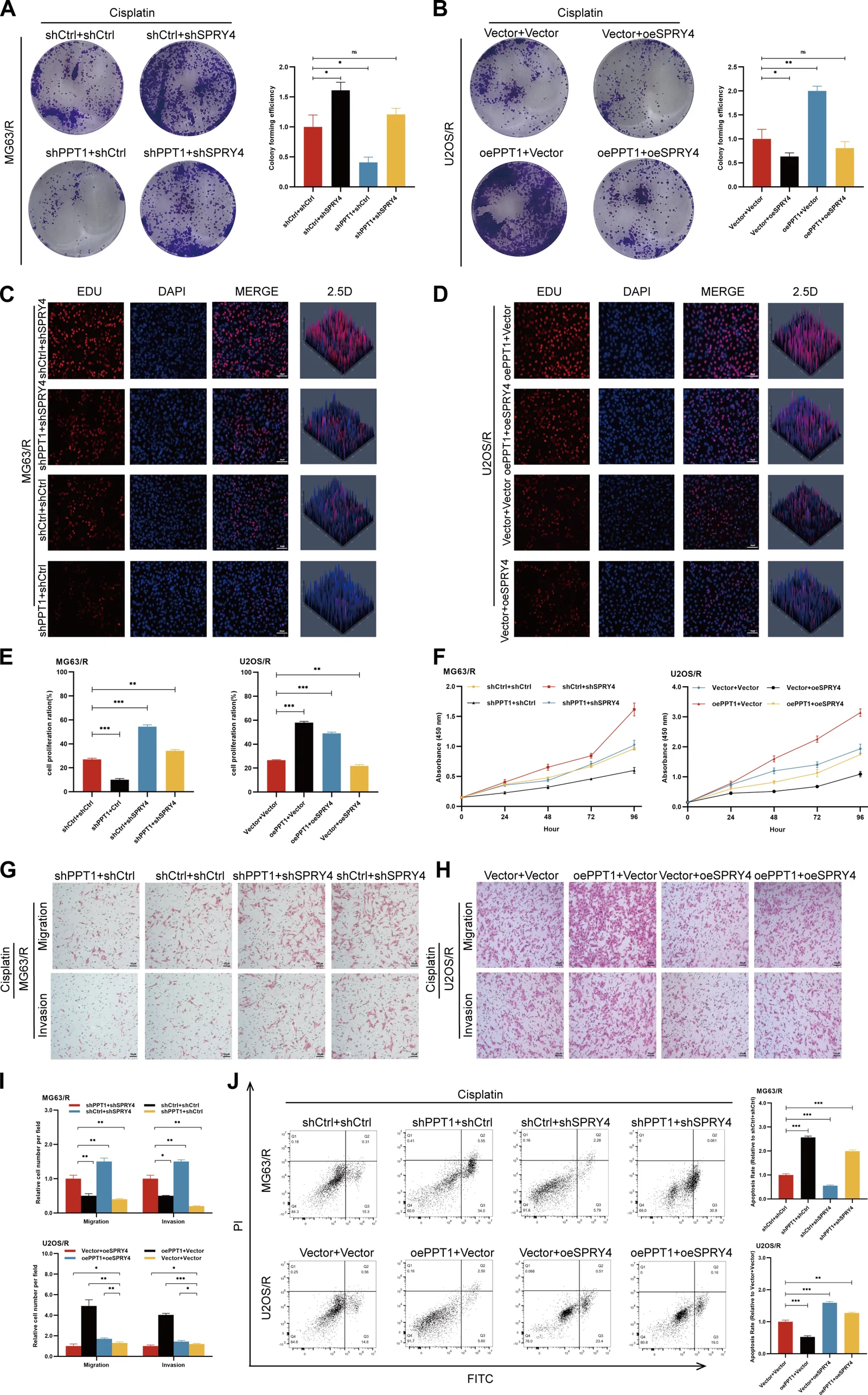
Regulation of OS cell proliferation, invasion, and apoptosis by PPT1 via SPRY4 palmitoylation. (A and B) Colony formation assays illustrating OS cell proliferation following SPRY4 knockdown in PPT1-knockdown MG63/R cells (A) and exogenous SPRY4 expression in PPT1-overexpressing U2OS/R cells (B). The left panels show representative images, and the right panels display quantitative analyses. (C to E) EdU incorporation assays assessing the impact of PPT1 knockdown or overexpression on the proliferation of MG63/R and U2OS/R cells. (C) and (D) present representative images, while (E) provides quantitative results (*n* = 3). Scale bar, 50 μm. (F) CCK-8 assay evaluating the effects of the interaction between PPT1 and SPRY4 on OS cell proliferation. (G to I) Migration and invasion assays demonstrating the influence of SPRY4 knockdown or overexpression in PPT1-knockdown or overexpressing MG63/R and U2OS/R cells. (G) and (H) present representative images, while (I) shows quantitative results (*n* = 3). Scale bar, 50 μm. (J) Flow cytometry analysis illustrating the effects of PPT1 and SPRY4 knockdown or overexpression on OS cell apoptosis. *P < 0.05, **P < 0.01, ***P < 0.001.

To assess the synergistic role of PPT1 and SPRY4 in OS cells, we knocked down SPRY4 in PPT1-knockdown MG63/R cells and overexpressed SPRY4 in PPT1-overexpressing U2OS/R cells, conducting a series of in vitro functional experiments. Colony formation assays revealed that PPT1 knockdown significantly inhibited cell proliferation, whereas SPRY4 knockdown partially reversed this effect. Conversely, exogenous SPRY4 expression in PPT1-overexpressing cells further promoted cell proliferation (Fig. 7A and B). EdU incorporation assays corroborated these findings, showing that PPT1 knockdown markedly reduced proliferative activity, which was partially alleviated by SPRY4 knockdown (Fig. 7C to E). Consistent results were observed in CCK-8 assays, highlighting the regulatory interplay between PPT1 and SPRY4 in cell proliferation (Fig. 7F).

Similarly, migration and invasion assays demonstrated that PPT1 knockdown markedly suppressed the migration and invasion abilities of MG63/R and U2OS/R cells, while SPRY4 knockdown partially restored these functions. In PPT1-overexpressing cells, SPRY4 overexpression further enhanced migration and invasion capabilities (Fig. 7G to I). Flow cytometry analysis further revealed that PPT1 knockdown increased OS cell apoptosis, whereas SPRY4 knockdown significantly reduced apoptosis induced by PPT1 knockdown. In contrast, PPT1 overexpression suppressed apoptosis, and SPRY4 overexpression further diminished apoptosis levels (Fig. 7J). Collectively, these findings suggest that PPT1 regulates proliferation, migration, invasion, and apoptosis in OS cells by modulating the palmitoylation status of SPRY4.

**Fig. 8.**
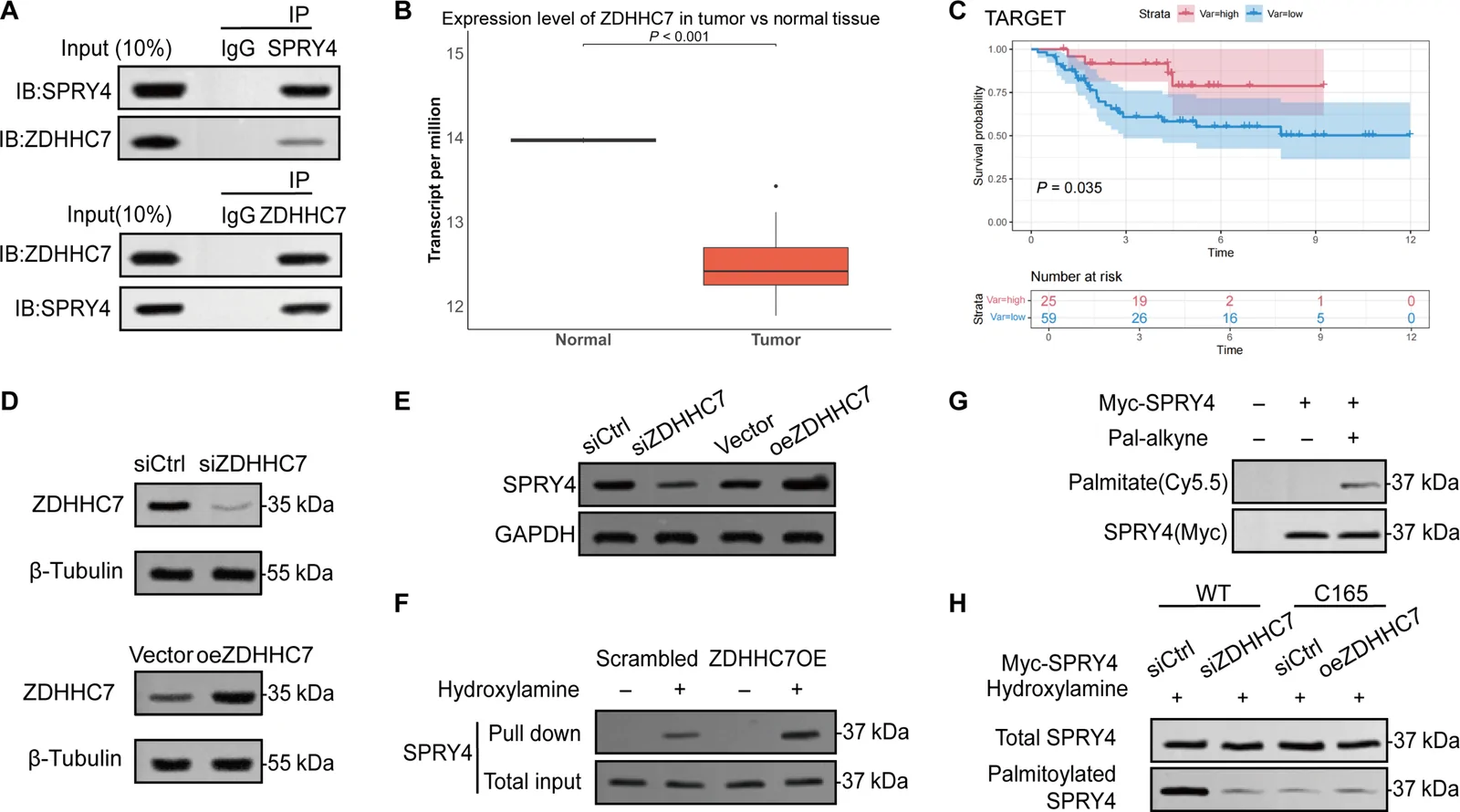
ZDHHC7 modulates MAPK signaling by palmitoylating SPRY4. (A) Co-immunoprecipitation assay demonstrating a direct interaction between SPRY4 and ZDHHC7. (B) Analysis of ZDHHC7 expression in OS and normal tissues based on the GSE14359 dataset. (C) Kaplan–Meier survival analysis of OS patients stratified by ZDHHC7 expression levels. (D) Western blot analysis of ZDHHC7 protein expression following knockdown or overexpression in MG63/R cells. (E) Western blot analysis of SPRY4 protein levels in MG63/R cells after ZDHHC7 knockdown or overexpression. (F) RAC assay showing a marked increase in SPRY4 palmitoylation upon ZDHHC7 overexpression in MG63/R cells. (G) Click chemistry assay further validating the increase in SPRY4 palmitoylation induced by ZDHHC7 overexpression. (H) Western blot analysis of SPRY4 palmitoylation in ZDHHC7-depleted cells and in cells expressing the SPRY4 C165 mutant following ZDHHC7 overexpression.

Meanwhile, we identified potential SPRY4-interacting proteins using Co-IP combined with mass spectrometry analysis (Fig. S5A and B). Given the known palmitoyltransferase activity of zinc finger DHHC-type palmitoyl transferase 7 (ZDHHC7) [15], we hypothesized that it may regulate SPRY4 activity via palmitoylation, thereby inhibiting the MAPK pathway. To investigate their interaction, we performed Co-IP assays and confirmed a direct interaction between SPRY4 and ZDHHC7 (Fig. 8A). Furthermore, analysis of the GSE14359 dataset revealed that ZDHHC7 expression was significantly lower in OS tissues compared to normal tissues (Fig. 8B). Kaplan–Meier survival analysis showed that high ZDHHC7 expression was significantly associated with improved prognosis in OS patients (Fig. 8C), suggesting a potential tumor-suppressive role for ZDHHC7 in OS development. Accordingly, we performed both knockdown and overexpression of ZDHHC7 in the MG63/R cell line (Fig. 8D). Notably, ZDHHC7 overexpression led to a significant increase in SPRY4 protein levels (Fig. 8E), while its mRNA levels remained unchanged (Fig. S5C), suggesting posttranscriptional regulation. In ZDHHC7-overexpressing MG63/R cells, RAC assays revealed a marked increase in SPRY4 palmitoylation, which was further validated by click chemistry analysis (Fig. 8F and G). Additionally, SPRY4 palmitoylation was significantly reduced following ZDHHC7 knockdown, similar to the C165 mutant. However, ZDHHC7 overexpression did not restore palmitoylation at the C165 site, indicating that C165 is likely the palmitoylation site on SPRY4 targeted by ZDHHC7 (Fig. 8H).

### Synergistic effect of GNS561 and cisplatin in OS

Ezurpimtrostat (GNS561), a targeted inhibitor of PPT1, has been widely utilized [20]. To investigate its potential as a therapeutic agent for OS, we conducted comprehensive in vitro and in vivo experiments to evaluate its effects on cell proliferation and apoptosis.

To determine the effective concentration of GNS561 in OS cells, we treated MG63/R and U2OS/R cell lines with various concentrations of GNS561 for 72 h and assessed proliferation inhibition using the CCK-8 assay. The results showed that GNS561 significantly suppressed OS cell proliferation, with IC_50_ values of approximately 1.0 μM in MG63/R cells and 1.2 μM in U2OS/R cells (Fig. S6). Based on these findings, 3 concentration groups were established: GNS561#1 (0.5 μM), GNS561#2 (1 μM), and GNS561#3 (2 μM), with a Dox-treated group serving as a control. CCK-8 assay and flow cytometry analyses demonstrated that GNS561 significantly inhibited cell proliferation and increased apoptosis in OS cells (Fig. S7A and B). Furthermore, caspase-3 activity was markedly elevated in the GNS561-treated group (Fig. S7C), indicating that the PPT1 inhibitor effectively induces apoptosis. To explore the potential clinical application of GNS561, we conducted combination treatment experiments with cisplatin. The CCK-8 assay revealed a pronounced synergistic effect of the GNS561 and cisplatin combination in suppressing cell proliferation (Fig. S7D). This finding was further supported by flow cytometry and caspase-3 activity assays, which showed a significant increase in apoptosis and caspase-3 activity following combination treatment (Fig. S7E and F).

**Fig. 9.**
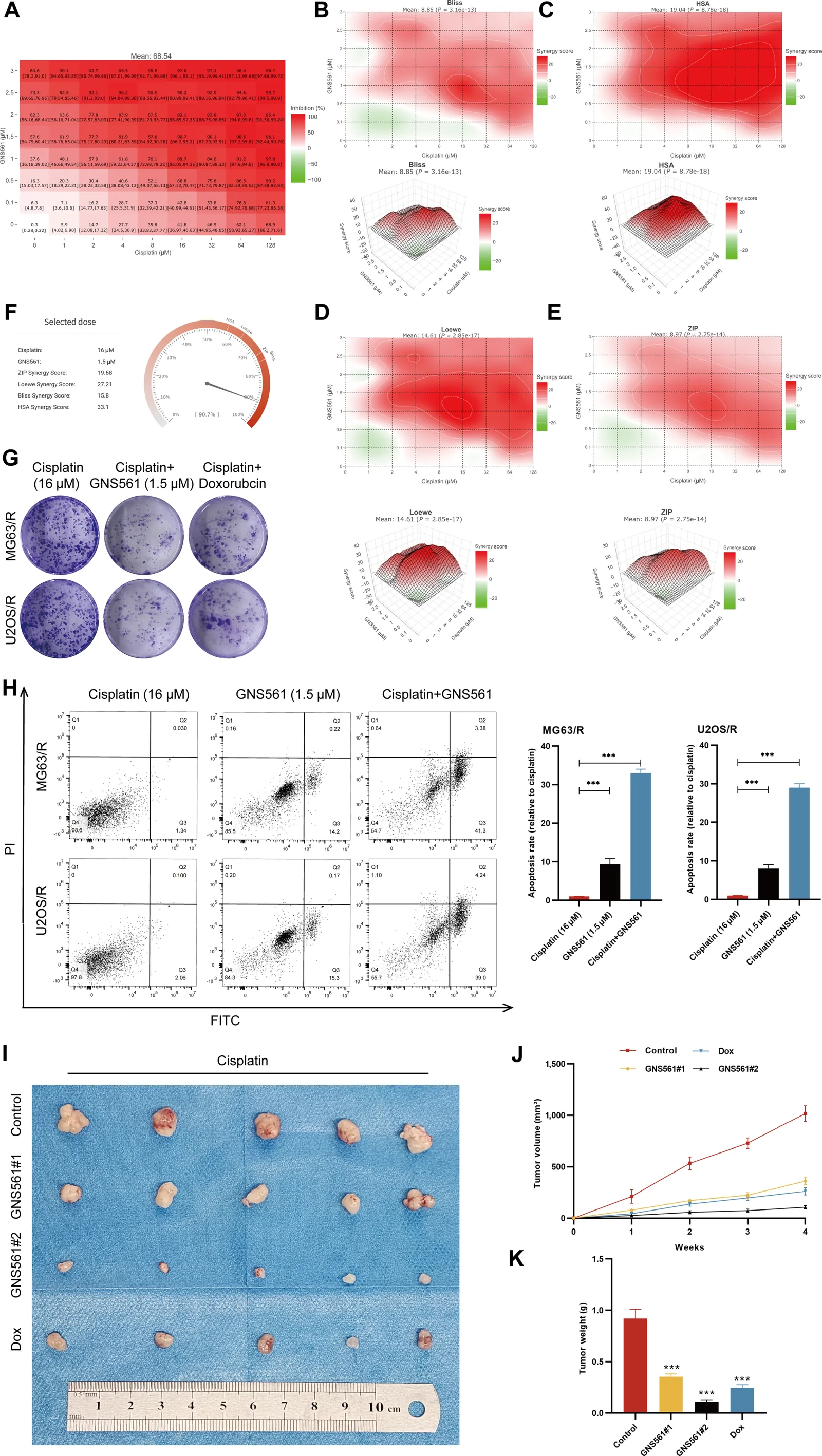
Visualization of the combination effects of GNS561 with cisplatin as a dual drug regimen. (A) Confidence interval graph for a pair of synergistic drugs (cisplatin and GNS561). (B to E) Synergy plots for the combined treatment of U2OS/R cells with cisplatin and GNS561 using Bliss (B), HSA (C), Loewe (D), and ZIP (E) methods. Synergy scores were calculated using the SynergyFinder software. Positive or negative synergy scores indicate synergistic or antagonistic effects, respectively. (F) Synergy measurement for a given dose combination of cisplatin with 1.5 μM GNS561. (G) Plate cloning formation experiment based on the recommended concentrations from the barometer in (F). (H) Flow cytometry and caspase-3 activity assays evaluating the apoptotic response to GNS561 and cisplatin combination treatment in MG63/R and U2OS/R cells. (I to K) Representative tumor images (I), tumor growth curves (J), and tumor weights (K) from nude mice across different treatment groups. ***P < 0.001.

To further quantify the synergistic effect of GNS561 and cisplatin, the 2 agents were co-administered, and their inhibitory effect on U2OS/R cells was assessed using the CCK-8 assay (Fig. 9A). SynergyFinderPlus software was subsequently used to evaluate drug synergy across various concentration combinations based on multiple mathematical models. The resulting synergy scores were: Bliss (8.85), highest single agent (HSA) (19.04), Loewe (14.61), and zero interaction potency (ZIP) (8.97). Despite minor variations across models, all consistently indicated a strong synergistic interaction between GNS561 and cisplatin, supporting the feasibility of their combined application (Fig. 9B to E).

To determine whether this synergy extends in vivo, we visualized inhibition rates across different drug concentrations alongside synergy scores calculated using the 4 models (Fig. S8). Drug combinations yielding inhibition rates above 90% were selected and presented in a radar plot (Fig. 9F). The observed inhibition rates substantially exceeded the values predicted by the HSA, Loewe, Bliss, and ZIP models, further validating the synergistic interaction between GNS561 and cisplatin. Of particular note, the combination of 16 μM cisplatin and 1.5 μM GNS561 achieved an inhibition rate exceeding 90%. This concentration pair was therefore selected for subsequent experiments aimed at elucidating the mechanisms underlying the synergistic effect.

Colony formation and flow cytometry assays revealed that combination treatment significantly suppressed tumor cell growth compared to either agent alone (Fig. 9G and H), underscoring the potential of GNS561 to enhance cisplatin efficacy in drug-resistant OS.

Furthermore, as shown in Fig. 9I to K, an OS xenograft model was established in nude mice for in vivo evaluation. Once tumors reached a predefined average volume, mice were randomly assigned to 4 groups: Control, Dox, GNS561#1, and GNS561#2, and treated for 4 weeks. Both GNS561#1 and GNS561#2 significantly reduced tumor volume and weight compared to the control group, and outperformed the Dox group, indicating that GNS561 exerts potent antitumor activity in vivo.

To assess the biosafety of GNS561 during treatment, histological examinations and liver and kidney function tests were conducted at the end of the study. H&E staining of major organs (heart, liver, spleen, lung, and kidney) from each mouse group revealed preserved tissue architecture without notable inflammatory cell infiltration, necrosis, or other pathological abnormalities (Fig. S9A), indicating that GNS561 did not cause significant organ toxicity. Furthermore, complete blood count and liver and kidney function analyses showed no signs of systemic toxicity in the GNS561#1 and GNS561#2 groups compared to the control group, further supporting the favorable in vivo safety profile of GNS561 (Fig. S9B).

**Fig. 10.**
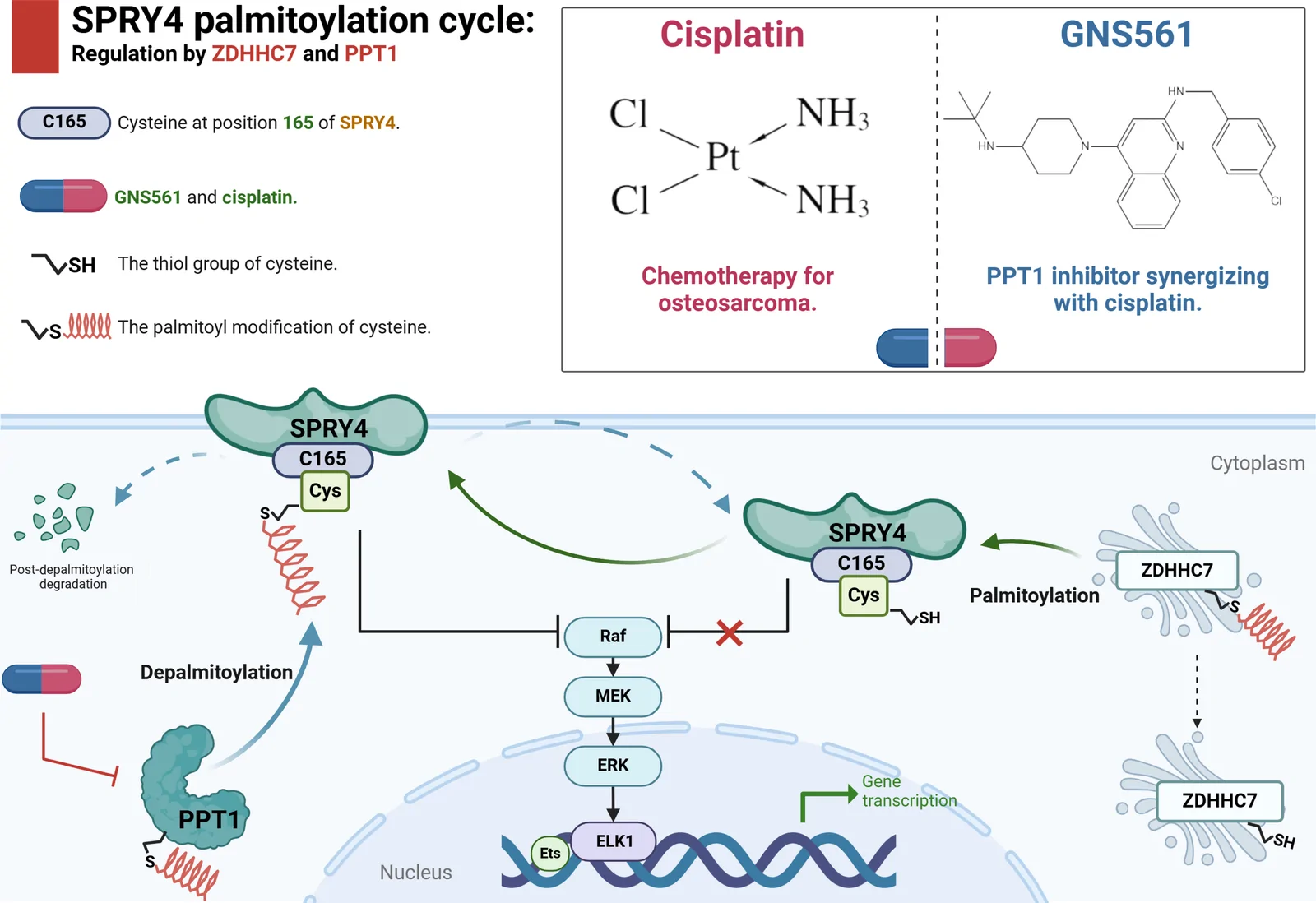
Schematic illustration of the palmitoylation cycle of SPRY4 and its regulatory mechanism in OS. ZDHHC7 catalyzes the palmitoylation of SPRY4 at cysteine 165 (C165), while PPT1 removes the palmitoyl group from this site, leading to SPRY4 degradation and establishing a dynamic “palmitoylation–depalmitoylation” cycle. Palmitoylated SPRY4 suppresses MAPK signaling, thereby regulating OS cell phenotypes such as proliferation, migration, and drug resistance. The PPT1 inhibitor GNS561 disrupts this regulatory cycle and enhances the antitumor efficacy of cisplatin when used in combination therapy.

The schematic model further illustrates the palmitoylation cycle of SPRY4 and its regulatory role in OS, highlighting the potential mechanism by which GNS561 modulates the MAPK signaling pathway via PPT1 inhibition (Fig. 10).

## Discussion

OS is a highly aggressive primary malignant bone tumor that primarily affects children and adolescents [1,2]. Despite advancements in treatment combining surgery and chemotherapy, such as cisplatin, the therapeutic efficacy remains markedly limited by the heterogeneity of OS and the development of cisplatin resistance [3,8]. This is particularly evident in cases of recurrence or metastasis, where patients exhibit poor prognoses and an event-free survival rate below 20% [2,21]. Although cisplatin is widely utilized in the treatment of various cancers [22–29], the underlying mechanisms of cisplatin resistance in OS are not yet fully understood [7,30–33]. Recent research indicates that protein depalmitoylation, mediated by depalmitoylating enzymes like PPT1, is crucial in regulating cell signaling, proliferation, and drug resistance by modulating protein stability and function, ultimately influencing tumor cell behavior [11,15]. In this study, we systematically investigated the role of PPT1 in OS and observed its elevated expression in OS tissues and cell lines. Our findings demonstrate that PPT1 activates the MAPK signaling pathway through depalmitoylation, thereby enhancing tumor cell proliferation and invasion while contributing to cisplatin resistance. Additionally, we validated the effectiveness of the PPT1 inhibitor GNS561 in vitro and in vivo, highlighting its potential to mitigate cisplatin resistance in OS. These findings provide valuable insights into the regulatory mechanisms of PPT1, suggesting novel therapeutic strategies and offering hope for improving the prognosis of OS patients.

Palmitoylation is a vital posttranslational modification that involves the attachment of palmitoyl groups to proteins, affecting their membrane localization, stability, and function. This modification plays a critical role in regulating cellular processes such as signaling, proliferation, and differentiation [15,34–44]. Conversely, depalmitoylation, the reverse process [45–48], is mediated by depalmitoylating enzymes like PPT1, which remove palmitoyl groups from proteins [49]. In this study, we integrated scRNA-seq and bulk sequencing (Bulk-seq) datasets to identify PPT1, a gene highly expressed in OS and linked to depalmitoylation. Using the OncoPredict algorithm, we uncovered a strong association between PPT1 expression and chemotherapy resistance. Experimental validation revealed that elevated PPT1 expression is closely related to tumor cell proliferation, migration, invasion, and drug resistance in OS. Further analyses, including GO, KEGG, GSVA, and GSEA, shed light on the potential functions of PPT1 in OS, emphasizing its roles in drug resistance, tumor-promoting responses, and cellular signaling regulation. Notably, PPT1’s significant enrichment in the MAPK signaling pathway highlights its aberrant activation and close association with tumor cell proliferation and survival.

To further elucidate the mechanism of PPT1, we investigated its potential interaction with SPRY4, a key negative regulator of the MAPK pathway [50–53]. Using various experimental approaches, we comprehensively analyzed how PPT1 regulates SPRY4 function via depalmitoylation, emphasizing the critical role of the C165 site in this process. Co-IP and mass spectrometry analyses confirmed the interaction between PPT1 and SPRY4, suggesting a functional association. Molecular docking simulations further supported this interaction and identified multiple potential binding sites. While these findings provided theoretical evidence, experimental validation of the key target sites was necessary. Accordingly, we conducted RAC and click chemistry experiments to assess PPT1’s regulatory effect on SPRY4 palmitoylation. The results showed that PPT1 knockdown significantly increased SPRY4 palmitoylation levels.

To identify the critical palmitoylation site of SPRY4, we constructed SPRY4 mutants at C165, C224, and C250. The results revealed that only the mutation at the C165 site significantly reduced SPRY4 palmitoylation levels, while mutations at the other sites had no comparable effect. Palmitoylation is an enzyme-mediated modification that typically occurs at cysteine (Cys) residues [14,15,54]. The mutation of the C165 site disrupts this specific modification site, preventing SPRY4 from undergoing palmitoylation at this position. PPT1 facilitates SPRY4 protein degradation through depalmitoylation, and the significant reduction in SPRY4 palmitoylation caused by the C165 mutation may impair its binding to PPT1 or alter target recognition. Without effective palmitoylation, PPT1 is unable to regulate SPRY4 protein stability, which may disrupt the MAPK signaling pathway. Notably, our study further revealed that SPRY4 stability is regulated by ZDHHC7. As a known palmitoyltransferase [15], ZDHHC7 directly interacts with SPRY4, as confirmed by Co-IP assays. Functional experiments showed that ZDHHC7 overexpression significantly enhanced SPRY4 palmitoylation, whereas ZDHHC7 knockdown markedly reduced this modification. Furthermore, in the C165 mutant, ZDHHC7 overexpression failed to restore SPRY4 palmitoylation, further confirming C165 as the critical site for this modification.

In summary, we propose a regulatory axis centered on SPRY4: ZDHHC7 promotes SPRY4 stabilization via palmitoylation, while PPT1 facilitates its degradation through depalmitoylation. This antagonistic regulation by ZDHHC7 and PPT1 collectively modulates MAPK pathway activation, thereby influencing OS development. Targeting SPRY4 palmitoylation—particularly at the critical C165 site—may represent a promising therapeutic strategy for MAPK-driven tumors and for overcoming cisplatin resistance.

GNS561 is a novel PPT1 inhibitor that has garnered significant attention for its potent antitumor activity across various cancers. By specifically targeting PPT1, GNS561 disrupts the autophagy pathway in tumor cells, leading to lysosomal membrane permeabilization [11,20,55–57]. This process ultimately inhibits tumor cell proliferation and survival by inducing apoptosis. Studies have highlighted the therapeutic potential of GNS561 in cancer treatment through mechanisms such as lysosomal dysfunction and zinc accumulation [20]. In preclinical studies, GNS561 has demonstrated remarkable antitumor activity in solid tumors, including hepatocellular carcinoma and cholangiocarcinoma, effectively reducing tumor volume and significantly delaying tumor progression. Its mechanisms of action include lysosomal dysfunction, zinc accumulation, mTOR pathway dysregulation, and autophagic flux blockade mediated by PPT1 inhibition [20,55]. With favorable pharmacokinetic properties and a strong safety profile, GNS561 has shown efficacy and tolerability in multiple clinical trials. Notably, phase I clinical trials for liver and biliary cancers have confirmed its safety and achieved tumor stabilization in some patients [20]. For OS, the PPT1 inhibitory effects of GNS561 have also demonstrated significant efficacy. Our in vitro and in vivo experiments revealed that GNS561 effectively inhibits OS cell proliferation and migration while inducing apoptosis, with the most pronounced effects observed at IC_50_ concentrations. Subsequent experiments showed a clear dose-dependent response, with higher concentrations of GNS561 enhancing tumor cell growth inhibition and apoptosis induction. Stronger antitumor effects at concentrations exceeding the IC_50_ further support GNS561’s potential as a candidate drug for OS treatment. Moreover, GNS561 exhibited synergistic effects when combined with cisplatin, significantly improving cisplatin efficacy in drug-resistant OS cells. This was evidenced by reduced colony-forming ability and increased apoptosis rates. These findings suggest that GNS561 modulates cisplatin resistance in OS cells through PPT1 inhibition. Our results indicate that combining GNS561 with cisplatin not only enhances the effectiveness of chemotherapy but also holds significant promise as a strategy to overcome cisplatin resistance.

This study systematically elucidates the pivotal role of PPT1 in the mechanisms underlying drug resistance in OS. We demonstrated that SPRY4 undergoes a dynamic palmitoylation cycle regulated by ZDHHC7 and PPT1, which modulates MAPK signaling and subsequently affects tumor cell proliferation, migration, apoptosis, and drug resistance. Experimental validation confirmed the efficacy of GNS561, a PPT1 inhibitor, in overcoming cisplatin resistance. GNS561 significantly inhibited OS cell proliferation and migration and, when combined with cisplatin, exhibited synergistic effects by enhancing cisplatin sensitivity in resistant cells and markedly increasing apoptosis rates. These findings highlight PPT1 as a critical factor in OS progression and drug resistance, providing a foundation for future therapeutic strategies targeting PPT1. GNS561 holds promise as an effective adjuvant therapy by enhancing cisplatin efficacy, overcoming resistance, and ultimately improving patient outcomes. Further research is warranted to explore the clinical potential of GNS561, focusing on validating its safety and efficacy in OS patients to optimize treatment strategies for this aggressive cancer.

## Materials and Methods

### Data collection

scRNA-seq data were retrieved from the GSE152048 [58] and GSE162454 [59] datasets, with GSE152048 including data from 11 OS patients and GSE162454 from 6 OS patients. Large-scale RNA sequencing data were primarily sourced from the TARGET-OS, GSE16091 [60], GSE39055 [61], and GSE21257 [62] datasets, which are part of the TARGET program and the Gene Expression Omnibus database.

### scRNA-seq analysis

The scRNA-seq data were processed using the “Seurat” R package (version 4.3.0). To ensure data quality, low-quality cells were filtered based on unique molecular identifier (UMI) counts, feature counts (nFeature), and mitochondrial gene proportions. Potential doublets and dead cells were excluded using the “DoubleFinder” package (version 2.0.3). Data normalization, scaling, and PCA were performed with the “LogNormalize” function. Cell clustering was performed using the “FindNeighbors” and “FindClusters” functions, and the distribution of cell clusters was visualized using UMAP and t-SNE.

### Gene enrichment analysis

GO analysis, KEGG analysis, and the identification and enrichment of differentially expressed genes were conducted using the “clusterProfiler” package (version 4.8.1). GSEA and GSVA were performed using the “GSEABase” (version 1.62.0) and “gsva” (version 1.48.1) R packages, respectively.

### Development of cisplatin-resistant OS cell lines

A cisplatin-resistant cell line, MG63/R and U2OS/R, was developed from the OS cell lines MG63 and U2OS by gradually increasing the cisplatin concentration. Initially, MG63 and U2OS cells were seeded in culture dishes and treated with 1 μM cisplatin (MCE, HY-17394) for 48 h. After cisplatin treatment, the cells were cultured in fresh medium until they recovered optimal growth. The cisplatin concentration was then gradually increased in 6 stages. Resistance was confirmed when cells grew stably at a concentration of 32 μM cisplatin [18,19]. Both MG63/R and U2OS/R cell lines were maintained in a humidified incubator at 37 °C with 5% CO₂ and were authenticated by short tandem repeat analysis.

### Molecular docking studies

The crystal structure of the target protein was retrieved from SWISS-MODEL and stored as a Protein Data Bank (PDB) file for subsequent analysis. Drug structures were obtained from the PubChem database in SDF format and converted to PDB format using Open Babel GUI software (version 3.1.1). Preprocessing and molecular docking were performed using PyMOL (version 2.5.7) and AutoDock4 (version 4.2.6). Preprocessing involved removing water molecules and adding nonpolar hydrogen atoms to both the protein and drug molecules. Molecular docking was carried out using AutoDock4, and the results were visualized with PyMOL for a comprehensive analysis. Effective docking was defined by a binding energy threshold of less than 2.0 kcal/mol.

### OncoPredict for drug sensitivity analysis

The OncoPredict package, an R tool for drug response prediction [18,63], was used to investigate the relationship between PPT1 expression and sensitivity to commonly used chemotherapy and molecular targeted drugs. An unpaired *t* test was applied to analyze the data, and a comprehensive evaluation of 198 drugs was conducted to assess sensitivity differences between the high-risk and low-risk groups. A significance threshold of *P* < 0.05 was adopted.

### siRNA and shRNA transfection

Two shRNA sequences targeting PPT1, 2 targeting SPRY4, and one siRNA sequence targeting ZDHHC7 were designed using the online tool provided by GenePharma (Shanghai, China). In the shRNA experiments, these sequences were cloned into the pLKO.1 vector. The pLKO.1 shRNA plasmids were co-transfected with packaging and envelope plasmids into human embryonic kidney 293T cells to produce lentiviral particles. OS cells in the logarithmic growth phase were seeded into 6-well plates and transduced with lentiviral particles when they reached 70% to 80% confluence. Polybrene was added to enhance transduction efficiency. After 12 h, the medium was replaced with fresh growth medium, and cells were incubated for an additional 24 h before being harvested. Knockdown efficiency was evaluated by quantitative real-time PCR (qRT-PCR). For the siRNA experiments, cells were transfected with Lipofectamine 3000 (Invitrogen) for 6 h. Following transfection, the medium was replaced with fresh complete culture medium, and cells were cultured for another 24 h before harvest. The sequences targeting PPT1, SPRY4, and ZDHHC7 are listed in Table S1.

### Resin-assisted capture

Forty-eight hours posttransfection, cells were harvested and lysed in a buffer containing 2% Triton X-100 and 0.2 mM hexadecylsulfonyl fluoride (HDSF) (Santa Cruz, Cat. No. sc-221708). Protein concentration was quantified using a bicinchoninic acid (BCA) protein assay kit (Thermo Fisher Scientific, Cat. No. 23227). Equal amounts of protein lysates were incubated with 2.5% sodium dodecyl sulfate (SDS) and 0.1% methyl methanethiosulfonate (MMTS) (Sigma, Cat. No. 64306) to block free thiol groups and prevent nonspecific binding in subsequent steps. The proteins were then precipitated, washed, and resuspended in binding buffer (100 mM HEPES, 1 mM EDTA, and 1% SDS, with protease inhibitors) before performing pull-down experiments using thiol-propyl-sepharose gel beads (GE Healthcare, Cat. No. 17-0420-01). The thiol groups in the resin specifically bind to palmitoyl ester bonds in the proteins, thereby enriching the palmitoylated proteins.

Palmitoylated proteins were eluted by incubating with neutral hydroxylamine (0.8 M, pH 7.4, Alfa Aesar, Cat. No. B22202), which cleaved the thioester bonds, releasing the palmitoylated proteins from the resin. The enriched proteins were further eluted with 50 mM DTT (dithiothreitol), denatured, and analyzed by Western blot.

### Click chemistry

Forty-eight hours posttransfection, cells were treated with culture medium containing 20 μM alkynyl-palmitic acid (Cayman Chemical, Cat. No. 13266) for 6 h to incorporate the alkyne group into the palmitoylated proteins, thereby providing reactive sites for subsequent click chemistry reactions. After treatment, the cells were washed with PBS and lysed using lysis buffer (50 mM Tris-HCl, pH 7.4, 150 mM NaCl, 1% Triton X-100, 1% SDS, and protease inhibitors) to release cellular proteins, including the target protein (SPRY4) and Myc-tagged PPT1. Protein concentration was determined using a BCA protein assay kit (Thermo Fisher Scientific, Cat. No. 23227).

Immunoprecipitation was performed using Myc-trap magnetic beads (Chromotek, Cat. No. ytma-10) to isolate Myc-tagged PPT1 from the protein mixture, yielding purified PPT1 for subsequent click chemistry reactions. The lysate was incubated with the beads at 4 °C for 2 h, followed by washing with lysis buffer to remove unbound proteins.

The immunoprecipitated proteins were incubated with click chemistry reagents (1 mM CuSO_4_, 1 mM TCEP, 0.1 mM TBTA, 40 μM azide-Cy5.5, and PBS, pH 7.4) at room temperature for 1 h, labeling the alkyne-containing palmitoylated proteins with a fluorescent group (e.g., Cy5.5). After labeling, the proteins were eluted from the beads and boiled in Laemmli sample buffer.

### Quantitative real-time PCR

When MG63/R and U2OS/R cells reached 80% confluence, they were harvested, and total RNA was extracted using TRIzol reagent (Invitrogen, USA). RNA was then reverse transcribed into cDNA using the PrimeScript RT reagent kit (TaKaRa Bio, Japan), following the manufacturer’s instructions. qRT-PCR amplification was performed using SYBR Premix Ex Taq II reagent (TaKaRa Bio, Japan) on the ABI 7500 Sequence Detection System. Data were analyzed using the 2^−ΔΔCT^ method, with glyceraldehyde-3-phosphate dehydrogenase (GAPDH) as the internal control. Primer sequences are provided in Table S2.

### Western blotting

Total protein was extracted from OS cell lysates and quantified using a BCA protein assay kit (Pierce, USA). Equal amounts of protein were resolved by 10% SDS-PAGE and transferred onto a polyvinylidene fluoride (PVDF) membrane. The membrane was blocked with Tris-buffered saline with Tween 20 (TBST) containing 5% skim milk at room temperature for 1 h, followed by incubation with the primary antibody at 4 °C overnight and the secondary antibody at room temperature for 2 h, as described in Table S3. Protein signals were detected using enhanced chemiluminescence reagents (Millipore) and captured with a chemiluminescence imaging system. Optical density was quantified and analyzed using ImageJ software.

### Immunofluorescence analysis

Apoptosis was evaluated using TUNEL staining, while PPT1 gene expression was analyzed via immunofluorescence. All experimental procedures were conducted following the manufacturer’s instructions (Beyotime, C1091). Cell nuclei were stained with DAPI and visualized using a laser confocal microscope.

### Cell viability assay and colony formation assay

EdU assay: Cells were incubated with 50 μM EdU labeling solution (Apexbio) for 2 h, followed by treatment with 500 μl of Cy3 azide click reaction mixture. The cells were incubated at room temperature in the dark for 30 min. After PBS washing, the cells were stained with 1 ml of Hoechst 33342 solution (5 μg/ml) for 30 min and visualized using a fluorescence microscope.

CCK-8 assay: MG63/R and U2OS/R cells were seeded into 96-well plates at a density of 5,000 cells per well. Before measurement, CCK-8 reagent (C0037, Beyotime) was added and incubated at 37 °C. Absorbance at 450 nm was measured every 24 h over a 72-h period.

Colony formation assay: MG63/R and U2OS/R cells were evenly seeded into 12-well plates at a density of 500 cells per well and cultured for 12 days, with medium changes performed regularly. The cells were fixed with formaldehyde, stained with crystal violet, and photographed. Colony numbers were analyzed using ImageJ software (version 1.8.0).

Soft agar colony formation assay: MG63/R and U2OS/R cells (5,000 cells per well) were suspended in 0.3% agar in Dulbecco’s modified Eagle medium (DMEM) and overlaid onto a 0.5% agar base layer. The cells were cultured at 37 °C for 2 weeks. Colonies were stained with 0.05% crystal violet, and those with a diameter >0.5 mm were counted. All experiments were performed in duplicate, with 3 technical replicates per condition.

### Migration and invasion assays

Migration assay: The migration capacity of OS cells was assessed using a Transwell assay. Cell suspensions were seeded into the upper chamber of the Transwell, while the lower chamber was filled with medium supplemented with 20% fetal bovine serum to serve as a chemoattractant. After 36 h of incubation at 37 °C in a 5% CO₂ environment, migrated cells were fixed with 4% paraformaldehyde, stained with crystal violet, and visualized under a microscope for imaging.

Invasion assay: The invasion ability of OS cells was evaluated using a Transwell assay with Matrigel-coated upper chambers. Cells suspended in basal medium were added to the upper chamber, and complete medium was placed in the lower chamber. After 36 h of incubation, noninvading cells in the upper chamber were removed, while the invaded cells were fixed with 4% paraformaldehyde, stained with crystal violet, and visualized under a microscope.

### Animal experiments

All animal experiments in this study were approved by the Experimental Animal Welfare Ethics Committee of Central South University (Approval No.: CSU-2024-0136). Six-week-old female BALB/c nude mice were obtained from Hunan SJA Laboratory Animal Co., Ltd. (Changsha, China). MG63/R cells (5 × 10^6^ cells per mouse) were subcutaneously injected into the right axillary region of mice in both experimental and control groups (*n* = 5 per group). Tumor size was measured regularly using calipers. When tumors reached the target average volume, cisplatin (3 mg/kg, twice weekly) was administered intraperitoneally at predetermined doses and intervals. To evaluate the in vivo inhibitory effects of GNS561 (MCE, HY-137978A) on tumor growth, mice in each group were intraperitoneally injected every 3 days with PBS (control group, shCtrl), GNS561#1 (15 mg/kg), GNS561#2 (30 mg/kg), or doxorubicin (0.5 mg/kg, MCE, HY-15142R). Tumor volume was calculated using the formula: (length × width^2^)/2. After the experiment, the mice were humanely euthanized, and their hearts, livers, spleens, lungs, and kidneys were harvested for blood sample analysis. Tumor tissues were also collected for further analyses, including H&E staining and evaluation of PPT1 and TUNEL expression.

### Mass spectrometry

Stably transfected MG63/R cells were treated with 10 μM of the proteasome inhibitor MG132 (MCE, HY-13259) for 4 h. Following cell lysis, immunoprecipitation was conducted using anti-Flag M2 affinity gel (Sigma-Aldrich, A2220). The immunoprecipitated proteins were separated by SDS-PAGE, and the resulting protein bands were excised and digested with trypsin. The digestion products were analyzed via liquid chromatography–tandem mass spectrometry (LC-MS/MS), and peptide identification was performed by Ollwegene Technology (Beijing, China).

### Co-immunoprecipitation

Total cellular protein was extracted using NP-40 lysis buffer supplemented with MG-132 (MCE, HY-13259; MCE, HY-K1002), and the supernatant was collected. The lysates were incubated with the specified antibody at 4 °C overnight, followed by the addition of protein A/G agarose beads and further incubation at 4 °C for 6 h. The immunoprecipitates were washed 3 times with lysis buffer and analyzed by Western blot. Antibodies used in the analysis are detailed in Table S3.

### MicroCT

After dissection, femurs and tibias were fixed in 4% paraformaldehyde solution and stored in PBS at 4 °C. High-resolution micro-CT (SkyScan 1276, SkyScan, Aartselaar, Belgium) was used to scan the specimens with a voxel size of 20.376 μm, a voltage of 100 kV, and a current of 200 μA. The region of interest was defined as 0.45 mm below the distal growth plate, extending 0.45 mm proximally, to analyze trabecular bone parameters such as BMD, BS, bone volume fraction (BV/TV), Tb.Th, Tb.N, and trabecular separation (Tb.Sp) [64]. Cortical bone parameters were also assessed, including total cross-sectional area within the periosteal envelope (Tt.Ar), Ct.Ar, Ct.Ar/Tt.Ar, and Ct.Th. These cortical parameters were measured over a 0.2-mm region at the mid-diaphysis of the femur [65].

### Evaluation of synergism using SynergyFinder plus software

Multiple models are employed to assess synergism, primarily in analyzing interactions among anticancer drugs. Utilizing models operating under different null hypotheses to analyze the same experimental data can lead to varying interpretations of the synergistic effects between the test drugs. To reliably evaluate the interactions between anti-OS drugs, we employed the SynergyFinder Plus calculator [66], which utilizes 4 main models for assessing synergism: ZIP, Loewe, Bliss, and HSA. The BLISS model assumes that the actions of the drugs are independent, and calculates the expected combination effect based on the probability of independent actions. The HSA model defines synergism as the response from a combination being greater than the response from either drug alone. The LOEWE model considers the dose–response relationship of each drug and calculates the expected additive response; if the response is higher than expected, it is marked as synergistic. The ZIP model evaluates the effect of a 2-drug combination, assuming that each drug does not affect the efficacy of the other.

### Statistical analysis

Data were analyzed using SPSS 21.0 and GraphPad 9.0. Results are expressed as mean ± standard deviation (SD). Group comparisons were performed using Student’s *t* test, one-way analysis of variance (ANOVA), or the Mann–Whitney *U* test, as appropriate. Survival analysis was conducted using the Kaplan–Meier method, with comparisons made via the log-rank test. A *P* value of <0.05 was considered statistically significant.

## Data Availability

scRNA-seq data were retrieved from the GSE152048 and GSE162454 datasets. Large-scale RNA sequencing data were primarily sourced from the TARGET-OS, GSE16091, GSE39055, and GSE21257 datasets.
