# Erratum to “Dual Regulation of Sprouty 4 Palmitoylation by ZDHHC7 and Palmitoyl-Protein Thioesterase 1: A Potential Therapeutic Strategy for Cisplatin-Resistant Osteosarcoma”

**DOI:** 10.34133/research.1379

**Published:** 2026-07-27

**Authors:** Tianlong Huang, Yifan Chen, Qiangqiang Zhao, Xin Wu, Hongxing Li, Xin Luo, Yang Su, Shengqun Zhang, Pan Liu, Ning Tang

**Affiliations:** ^1^ Orthopaedic Department, The Second Xiangya Hospital of Central South University, Changsha, Hunan, China.; ^2^ Department of Hematology, Liuzhou People’s Hospital affiliated to Guangxi Medical University, Liuzhou, Guangxi, China.; ^3^ Department of Hematology, The Qinghai Provincial People’s Hospital, Xining, Qinghai, China.; ^4^ Department of Spine Surgery, Third Xiangya Hospital, Central South University, Changsha, Hunan, China.; ^5^ Department of Orthopaedic, The Central Hospital of Shaoyang, Shaoyang, China.; ^6^ Changsha Medical University, Changsha, Hunan, China.; ^7^ Orthopaedic Department, The Third Xiangya Hospital of Central South University, Changsha, Hunan, China.

In the Research Article “Dual Regulation of Sprouty 4 Palmitoylation by ZDHHC7 and Palmitoyl-Protein Thioesterase 1: A Potential Therapeutic Strategy for Cisplatin-Resistant Osteosarcoma”, the authors have identified an error in Fig. 7. The image used in panel G was incorrect. This error does not affect the results or the main conclusions of the study, but the authors sincerely apologize for any inconvenience it may have caused [1].

The corrected Fig. 7 is provided below, and it has also been corrected in the PDF and HTML.

**Fig. 7.**
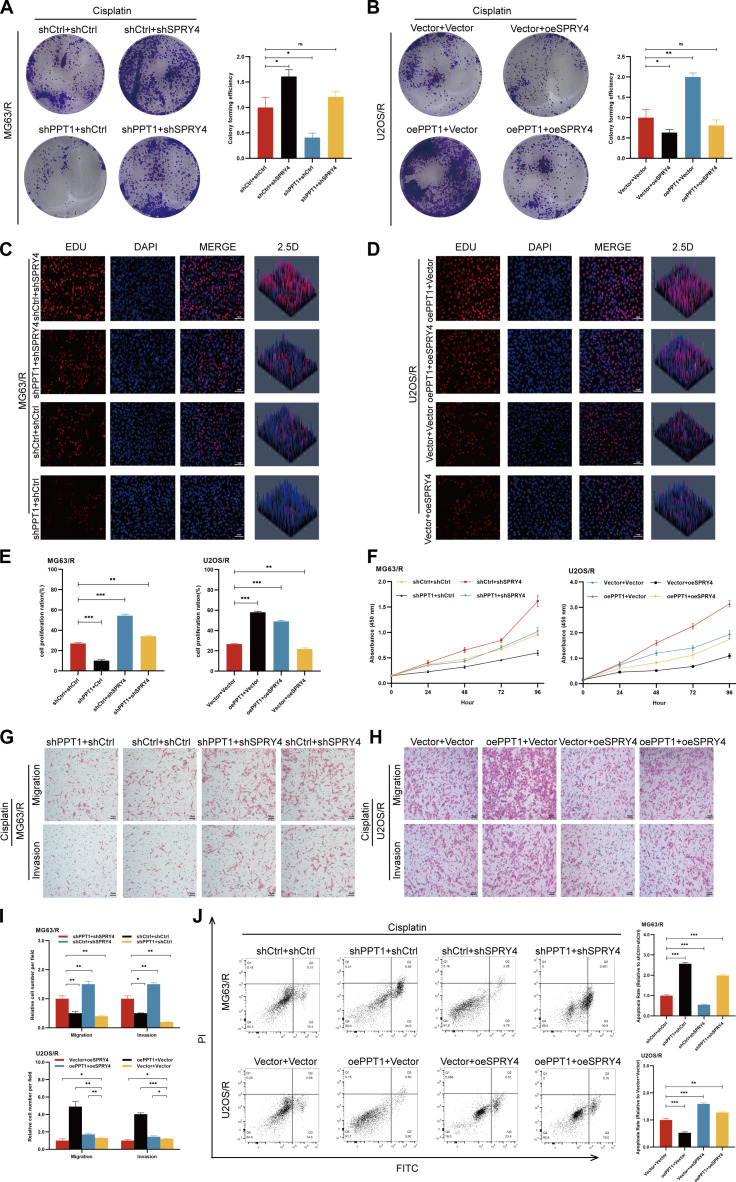
Regulation of OS cell proliferation, invasion, and apoptosis by PPT1 via SPRY4 palmitoylation. (A and B) Colony formation assays illustrating OS cell proliferation followingSPRY4 knockdown in PPT1-knockdown MG63/R cells (A) and exogenous SPRY4 expression in PPT1-overexpressing U2OS/R cells (B). The left panels show representative images, and the right panels display quantitative analyses. (C to E) EdU incorporation assays assessing the impact of PPT1 knockdown or overexpression on the proliferation ofMG63/R and U2OS/R cells. (C) and (D) present representative images, while (E) provides quantitative results (*n* = 3). Scale bar, 50 μm. (F) CCK-8 assay evaluating the effects of the interaction between PPT1 and SPRY4 on OS cell proliferation. (G to I) Migration and invasion assays demonstrating the influence of SPRY4 knockdown or overexpression in PPT1-knockdown or overexpressing MG63/R (G) and U2OS/R (H) cells. (G) and (H) present representative images, while (I) shows quantitative results (*n* = 3). Scale bar, 50 μm. (J) Flow cytometry analysis illustrating the effects of PPT1 and SPRY4 knockdown or overexpression on OS cell apoptosis. *P < 0.05, **P < 0.01, ***P < 0.001.
