# Supplementary material for: Dual Regulation of Sprouty 4 Palmitoylation by ZDHHC7 and Palmitoyl-Protein Thioesterase 1: A Potential Therapeutic Strategy for Cisplatin-Resistant Osteosarcoma
Source: Research (Wash D C). 2025 May 23;8:0708. doi: 10.34133/research.0708 (PMC12099059; doi:10.34133/research.0708)
Supplement: Supplementary 1 — Figs. S1 to S9 Tables S1 to S3 [file research.0708.f1.zip › Supplementary Information.docx]

# **Supplementary Materials**

**Supplementary Figures**


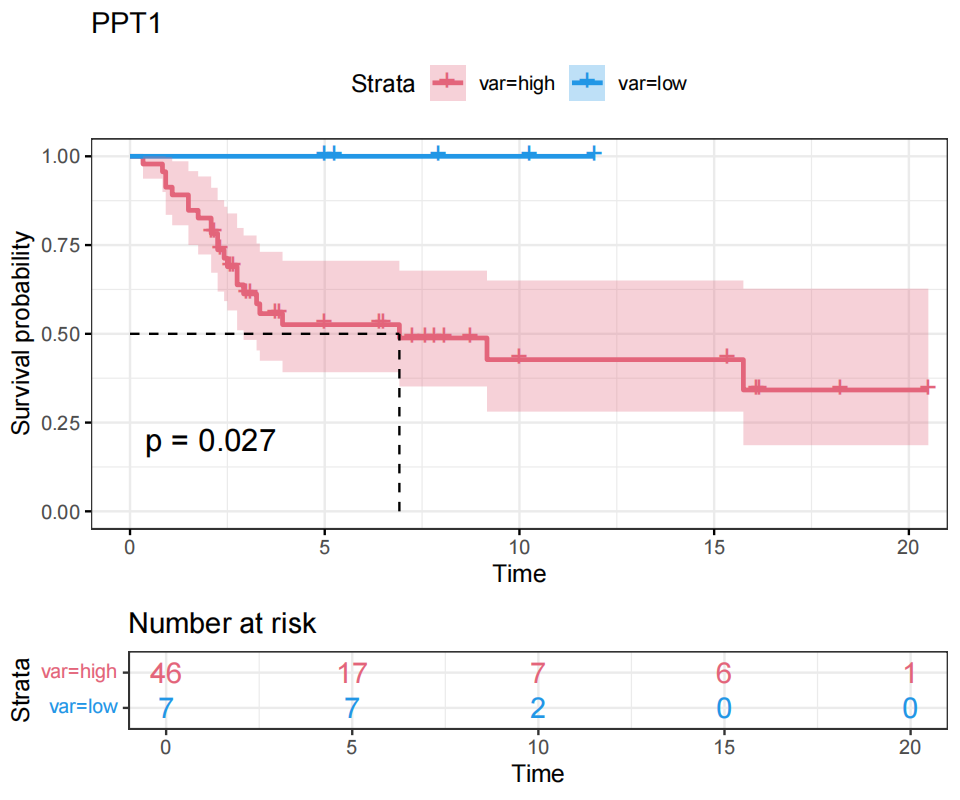


**Figure S1. Kaplan-Meier Survival Analysis of PPT1 in the GSE21257 Dataset.**


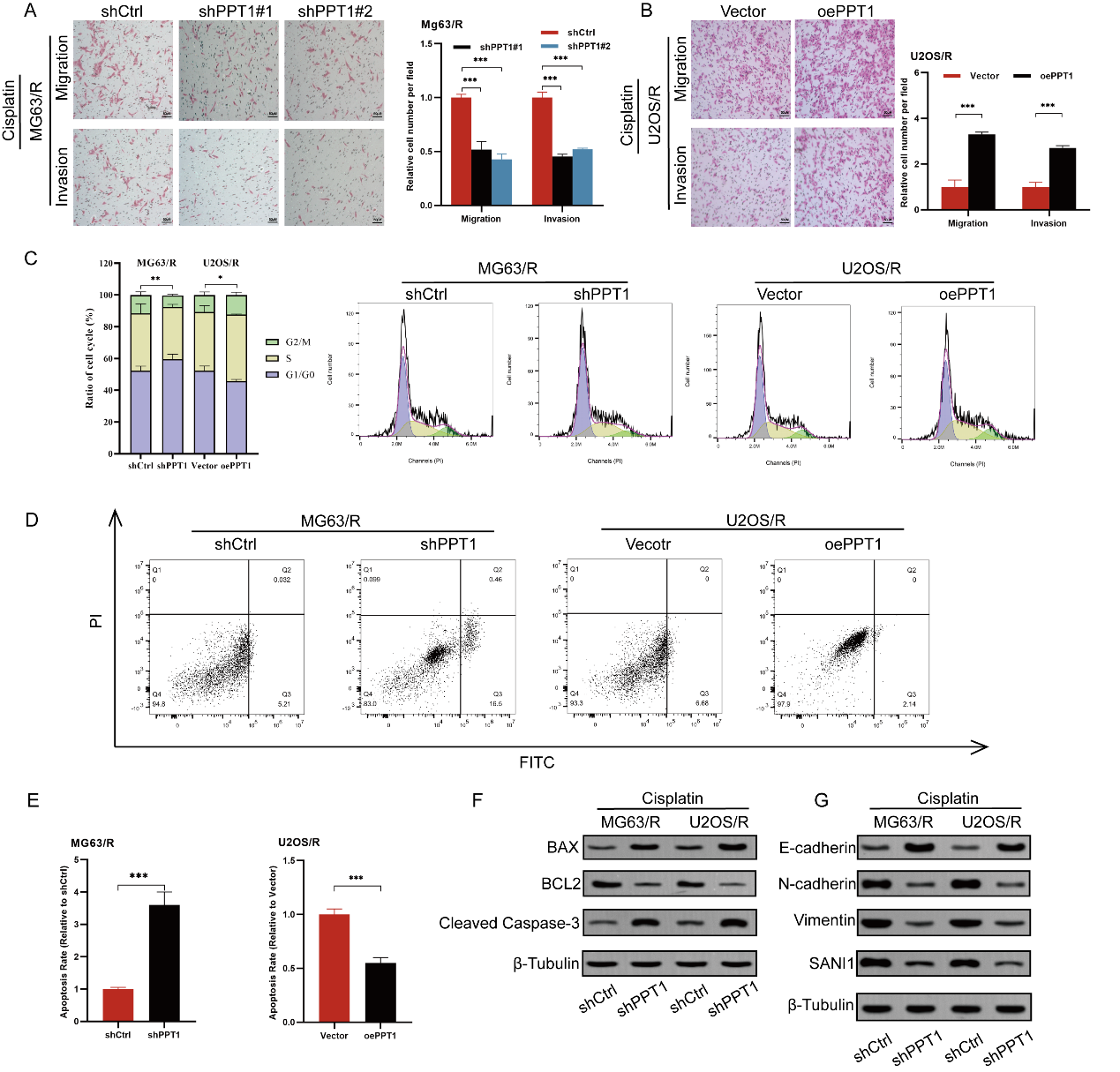


**Figure S2. Effects of PPT1 on Migration, Invasion, and Apoptosis of Osteosarcoma Cells In Vitro.** (A-B) Migration and invasion assays demonstrating the impact of PPT1 knockdown or overexpression on the migration and invasion capabilities of MG63/R and U2OS/R cells. Representative images are shown in the left panel, while statistical analyses are presented in the right panel. Scale bar, 50 μm. (C) Flow cytometry analysis of cell cycle distribution following PPT1 knockdown or overexpression in MG63/R and U2OS/R cells. (D-E) Flow cytometry analysis and corresponding quantitative evaluation of apoptosis levels in MG63/R and U2OS/R cells after PPT1 knockdown or overexpression. (F) Western blot analysis of apoptosis-related proteins (BAX, BCL2, Cleaved Caspase-3), illustrating the role of PPT1 in regulating the apoptotic process. (G) Western blot analysis of EMT markers (E-cadherin, N-cadherin, Vimentin, SNAI1) to investigate the effects of PPT1 on epithelial-mesenchymal transition (EMT) in osteosarcoma cells.


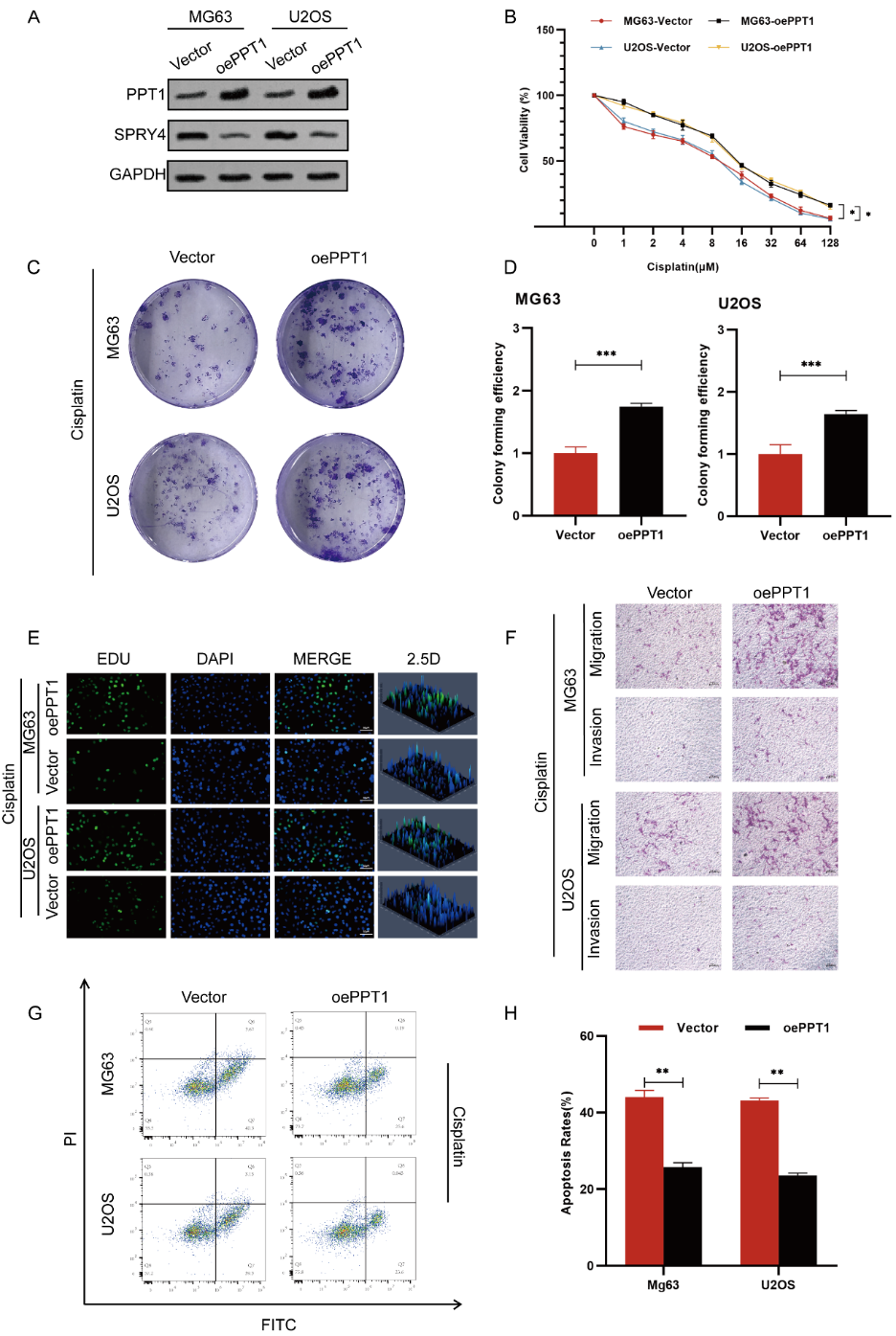


**Figure S3. Effects of PPT1 Overexpression in Parental Osteosarcoma Cells.** (A) Western blot analysis of PPT1 and SPRY4 protein expression following PPT1 overexpression in MG63 and U2OS cells. (B) Cell viability of osteosarcoma cells treated with various concentrations of cisplatin, as determined by the CCK-8 assay (n = 3). (C) Representative images from colony formation assays in MG63 and U2OS cells after PPT1 overexpression. (D) Quantitative analysis of colony formation efficiency in MG63 and U2OS cells following PPT1 overexpression. (E) EdU incorporation assays assessing the proliferation of MG63 and U2OS cells upon PPT1 overexpression. Scale bar, 50 μm. (F) Migration and invasion assays evaluating the effects of PPT1 overexpression on the motility and invasiveness of MG63 and U2OS cells. Scale bar, 200 μm. (G–H) Flow cytometry analysis and quantification of apoptosis in MG63 and U2OS cells after PPT1 overexpression.


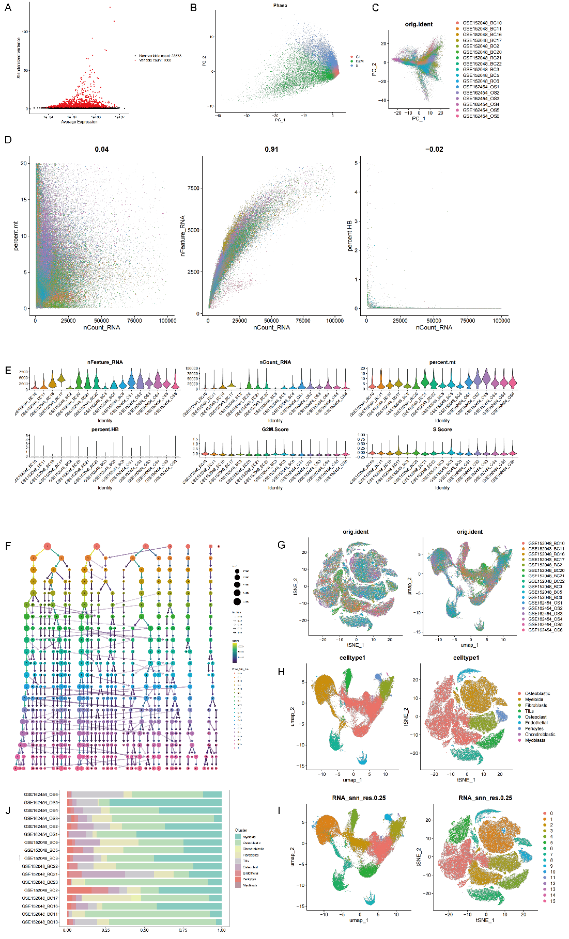


**Figure S4. Quality Control and Analysis of Single-Cell RNA Sequencing Data.** (A) Scatter plot showing the average gene expression versus standardized variance. Red points indicate variable genes (3,000), while black points represent non-variable genes (23,578). High-variance genes are selected for further analysis. (B) Principal component analysis (PCA) of cell cycle stages. Each point corresponds to an individual cell, with color coding representing the respective cell cycle phase (G1, G2M, S). (C) Principal component analysis (PCA) of the sample data. (D) Scatter plot displaying RNA counts and quality control metrics post-filtering. *nCount_RNA* denotes the total RNA molecules detected in each cell, *percent.mt* indicates the proportion of mitochondrial genes, *nFeature_RNA* represents the number of distinct genes detected per cell, and *percent.HB* refers to the proportion of hemoglobin genes. (E) Violin plot illustrating the distribution of quality control metrics across different sample groups. Metrics include *nFeature_RNA* (number of detected features), *nCount_RNA* (total RNA count), *percent.mt* (percentage of mitochondrial genes), *percent.HB* (percentage of hemoglobin genes), G2M.Score, and S.Score. (F) Clustree plot depicting cell clustering at different resolutions. Each node represents a cluster, with node size proportional to cell count, and color indicating the resolution used to determine the optimal clustering. (G) t-SNE and UMAP plots, with color indicating distinct sample groups. These plots highlight differences and consistencies between sample groups. (H-I) t-SNE and UMAP plots, with color representing different cell types. These plots demonstrate clustering results along with cell type information. J: Bar chart showing the proportion of each cell type across different sample groups. The colors represent distinct cell types.


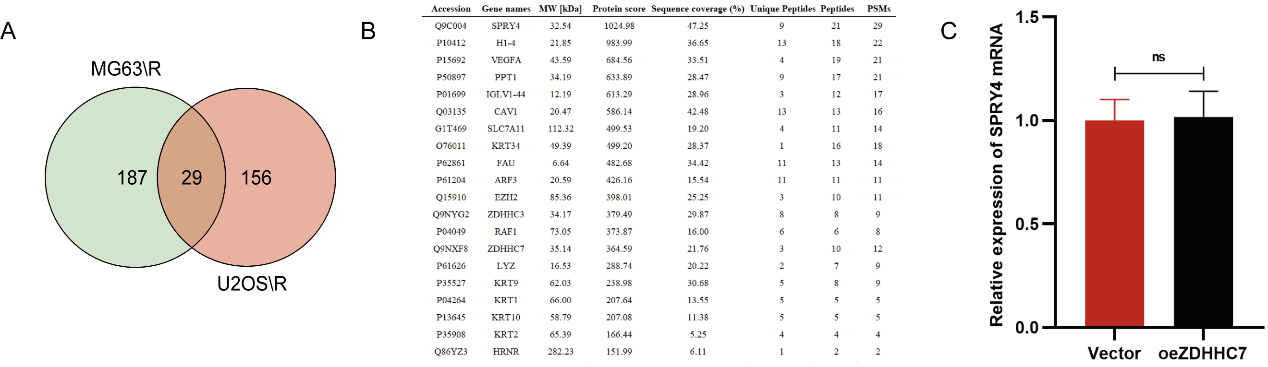


**Figure S5.** (A) Venn diagram of SPRY4-interacting proteins identified through liquid chromatography-tandem mass spectrometry (LC-MS/MS) in MG63/R and U2OS/R cells, with overlapping regions indicating common interacting proteins. (B) List of potential SPRY4-interacting proteins identified by mass spectrometry in MG63/R and U2OS/R cell lines. (C) Relative mRNA expression levels of SPRY4 after ZDHHC7 overexpression.


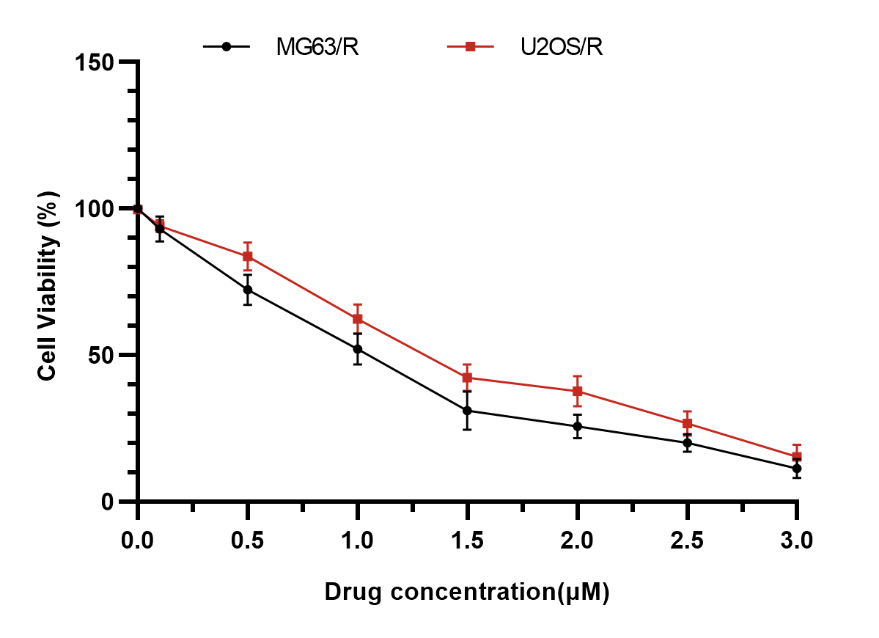


**Figure S6. Dose-Response Curve of GNS561 in MG63/R and U2OS/R Osteosarcoma Cell Lines.** MG63/R and U2OS/R cells were treated with varying concentrations of GNS561 (0, 0.1, 0.5, 1, 1.5, 2, 2.5, 3 μM) for 72 hours, and cell viability was measured using the CCK-8 assay. The results demonstrate that GNS561 reduces cell viability in both cell lines in a concentration-dependent manner. Data are presented as the mean ± standard deviation (SD) from three independent experiments. The IC50 values of GNS561 were approximately 1.0 μM in MG63/R cells and 1.2 μM in U2OS/R cells.


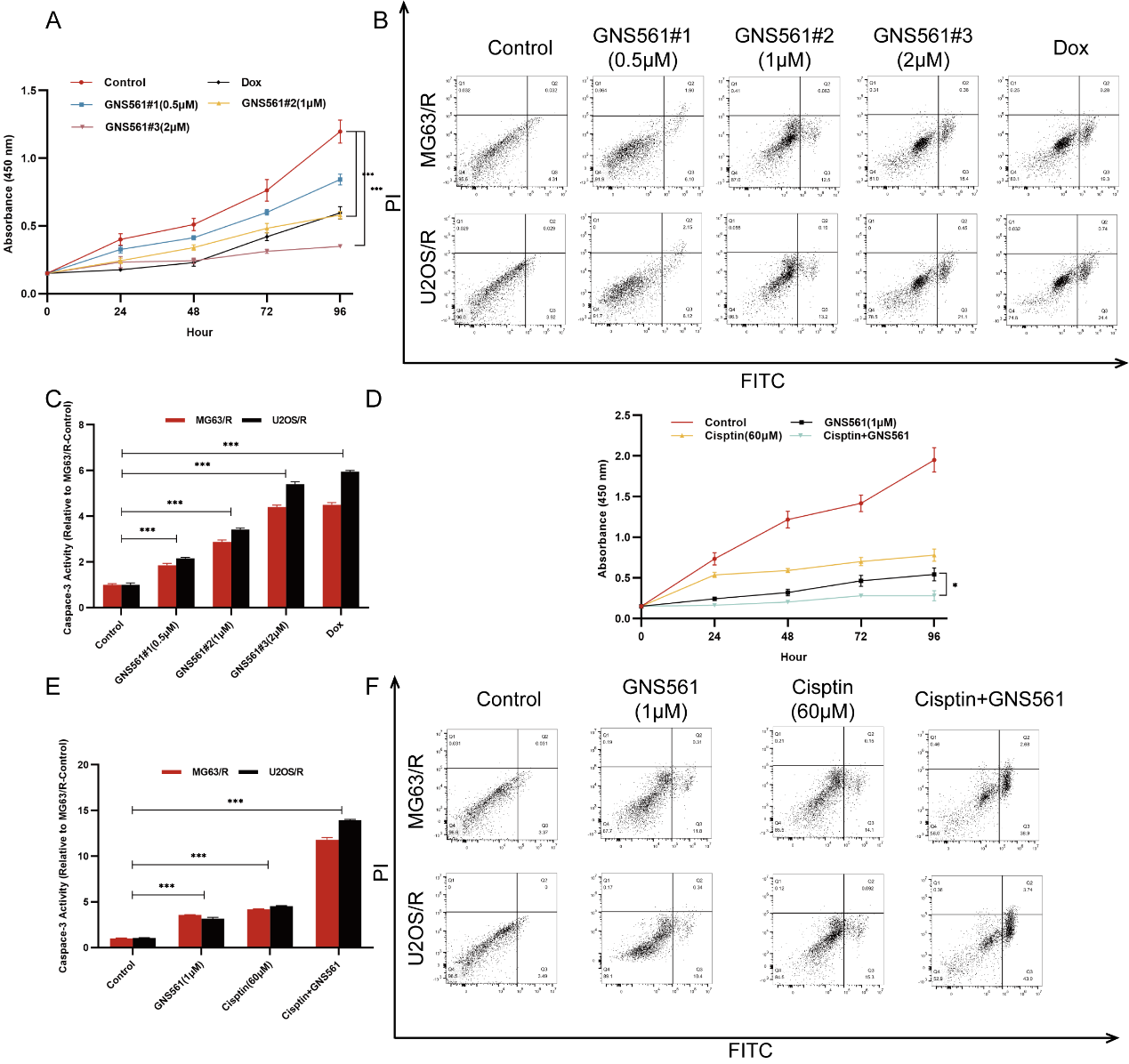


**Figure S7. Effects of the Inhibitor on In Vitro Cell Proliferation, Migration, In Vivo Tumor Growth, and Mechanisms.** (A) CCK-8 assay showing the effects of various concentrations of GNS561 and the Dox control group on the proliferation of MG63/R and U2OS/R cells. (B) Flow cytometry analysis depicting apoptosis levels in MG63/R and U2OS/R cells treated with different concentrations of GNS561 and the Dox control group. (C) Caspase-3 activity assay quantifying apoptosis in MG63/R and U2OS/R cells after treatment with various concentrations of GNS561 and the Dox control group. (D) CCK-8 assay assessing the impact of GNS561 combined with cisplatin on the proliferation of MG63/R and U2OS/R cells. (E) Flow cytometry analysis illustrating apoptosis levels in MG63/R and U2OS/R cells following combined treatment with GNS561 and cisplatin. (F) Caspase-3 activity assay evaluating apoptosis levels in MG63/R and U2OS/R cells treated with GNS561 and cisplatin in combination. (G) Representative tumor images from different treatment groups in animal experiments. (H) Tumor growth curves for animals in various treatment groups. (I) Comparison of tumor weights among the treatment groups.


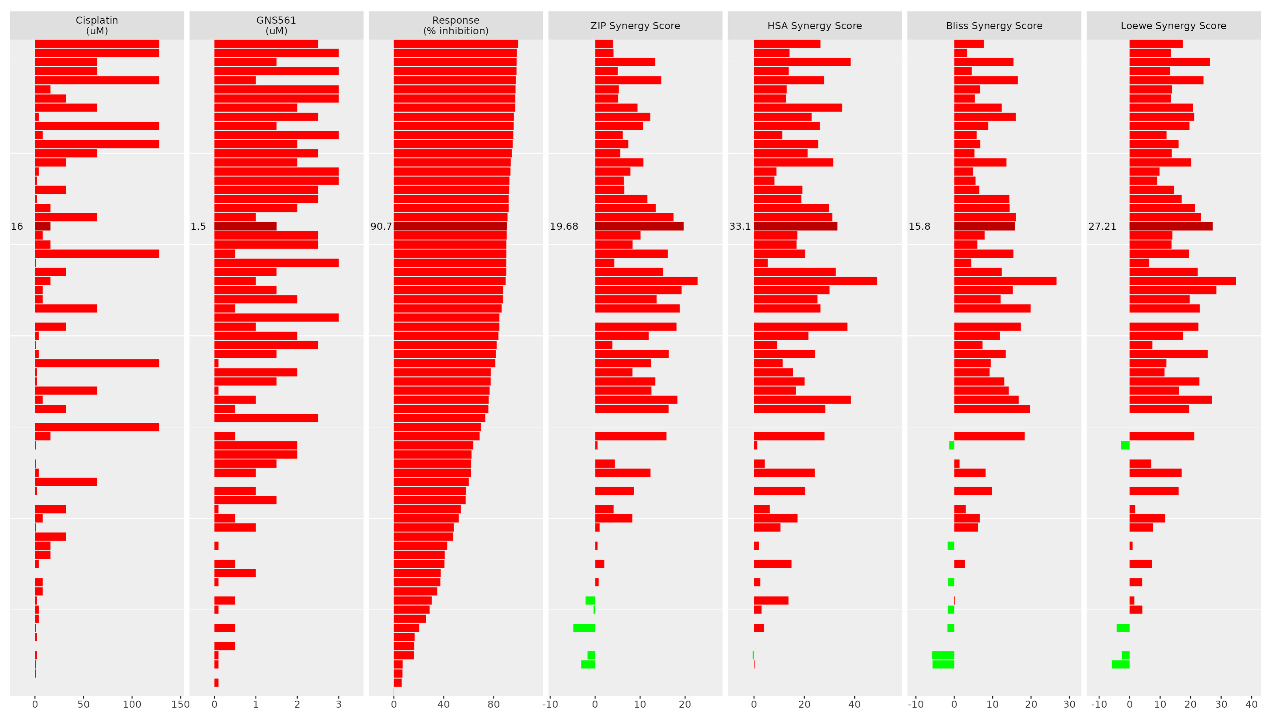


**FigureS8. Drug concentrations and synergy scores for groups where the inhibitory response exceeded 90 %.**


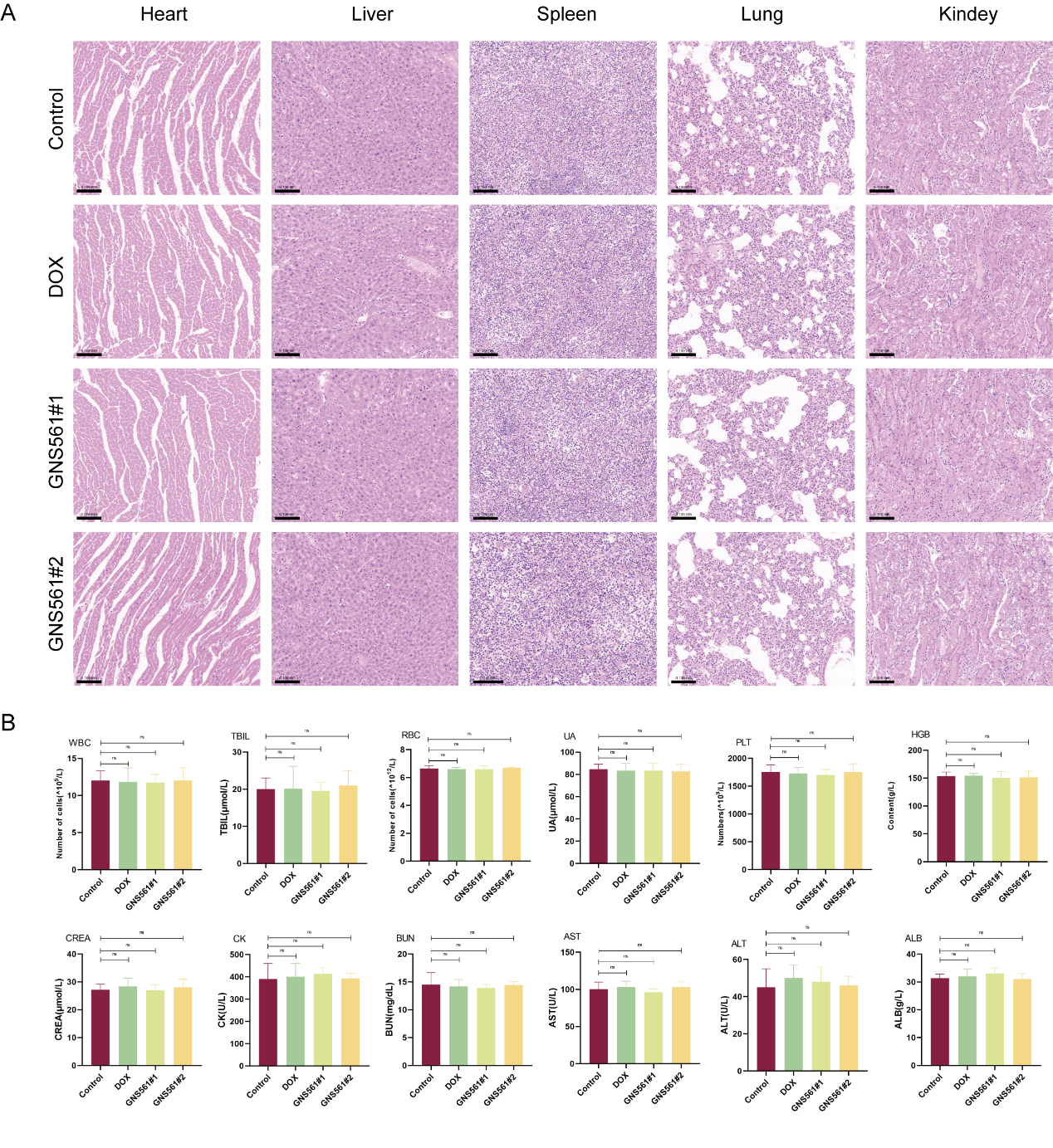


**Figure S9. Safety evaluation of GNS561 during in vivo treatment.** (A) H&E staining analysis of major organs (heart, liver, spleen, lungs, kidneys) from mice in various treatment groups (control, DOX, GNS561#1, and GNS561#2) (scale bar: 100 μm). (B) Blood biochemical analysis, including routine blood tests and liver and kidney function markers (n = 5).

**Supplementary Tables**

**Table S1** siRNA and shRNA Sequences Targeting PPT1, SPRY4, and ZDHHC7

| Name |  |  |
| --- | --- | --- |
| Homo-PPT1-shRNA-1 |  | 5’-GCACTTGCTAAGGATCCTAAA-3’ |
|  |  |  |
| Homo-PPT1-shRNA-2 |  | 5’-GCACTTGCTAAGGATCCTAAA-3’ |
|  |  |  |
| Homo-SPRY4-shRNA-1 |  | 5’-GCACTTGCTAAGGATCCTAAA-3’ |
|  |  |  |
| Homo-SPRY4-shRNA-2 |  | 5’-CCCAGACTCTGGTCAACTATG-3’ |
|  |  |  |
| Homo-ZDHHC7-siRNA-1 |  | 5’-UUUCGUAGCGUUUCCUUUGGG-3’ |

**Table S2** Primer Sequences Used for qRT-PCR

| Name | Sequences |  |
| --- | --- | --- |
| PPT1-1 | PCR primer F | 5’-TGTTTTGTGGACCTCCCGATG-3’ |
|  | PCR primer R | 5’-CATGCCAGGCACCCGTCTGC-3’ |
| PPT1-2 | PCR primer F | 5’-AGCCCAAGTGACTTGACC-3’ |
|  | PCR primer R | 5’-TTGATCACAGCGTTGATTT-3’ |
| SPRY4 | PCR primer F | 5’-TCTGACCAACGGCTCTTAGAC-3’ |
|  | PCR primer R | 5’-GTGCCATAGTTGACCAGAGTC-3’ |

**Table S3** Antibody Information Used in This Study

| Antibody name | item number and supplier |  |
| --- | --- | --- |
| PPT1 | ab89022  abcam |  |
| SPRY4 | 22765-1-AP |  |
|  | Proteintech |  |
| ZDHHC7 | ab138210 |  |
|  | abcam |  |
| E- cadherin | 20874-1-AP |  |
|  | Proteintech |  |
| N-cadherin | 22018-1-AP |  |
|  | Proteintech |  |
| Vimentin | #5741 |  |
|  | CST |  |
| beta-tubulin | sc-5274 |  |
|  | Santa Cruz Biotechnology |  |
| Bcl-2 | ab32124 |  |
|  | Abcam |  |
| Cleaved Caspase-3 | ab2302 |  |
|  | Abcam |  |
| BAX | ab32503 |  |
|  | Abcam |  |
