# Supplementary figures and images for: Dual Regulation of Sprouty 4 Palmitoylation by ZDHHC7 and Palmitoyl-Protein Thioesterase 1: A Potential Therapeutic Strategy for Cisplatin-Resistant Osteosarcoma
Source: Research (Wash D C). 2025 May 23;8:0708. doi: 10.34133/research.0708 (PMC12099059; doi:10.34133/research.0708)

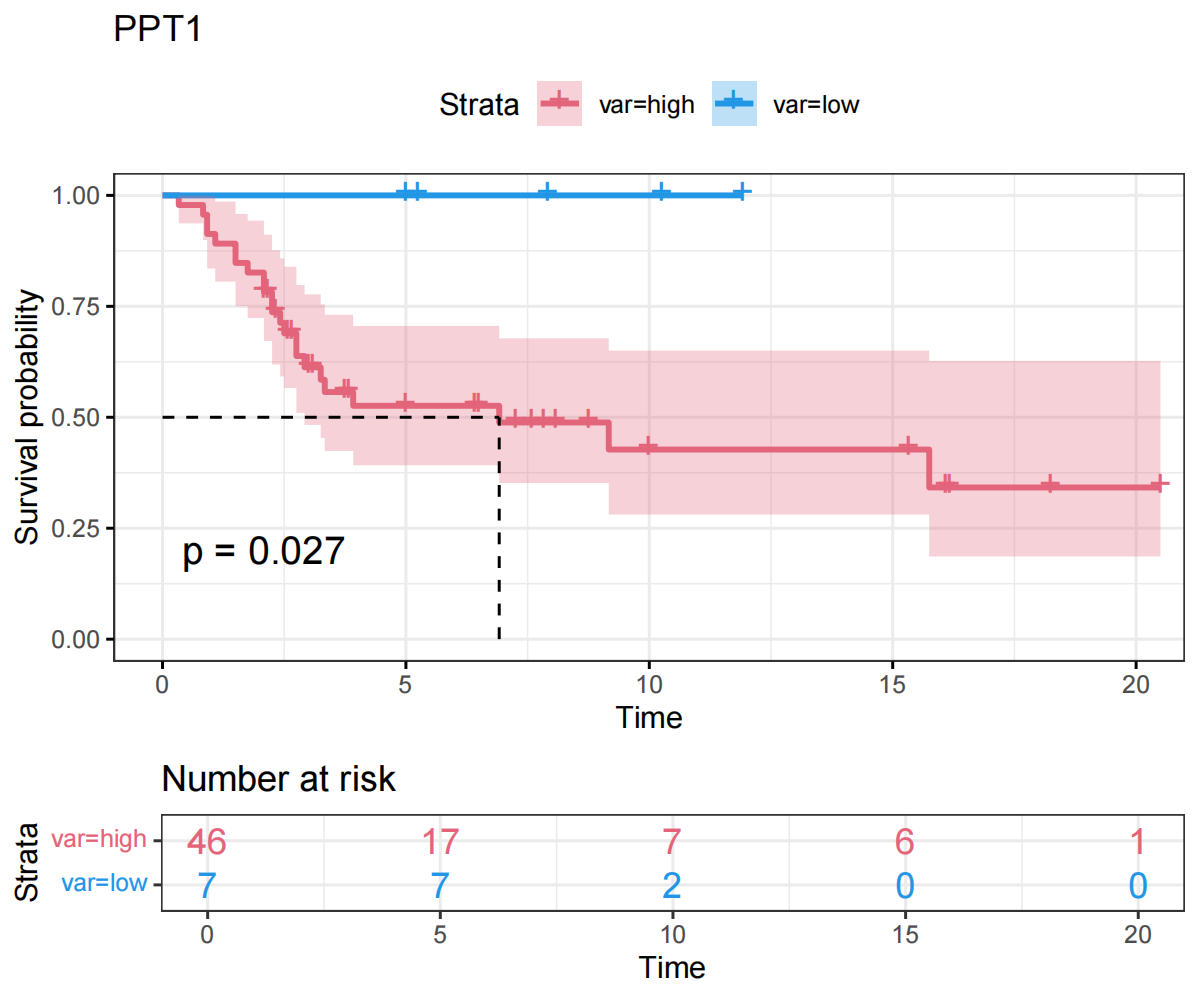

Supplement: Supplementary 1 — Figs. S1 to S9 Tables S1 to S3 [file research.0708.f1.zip › Figure S1.png]

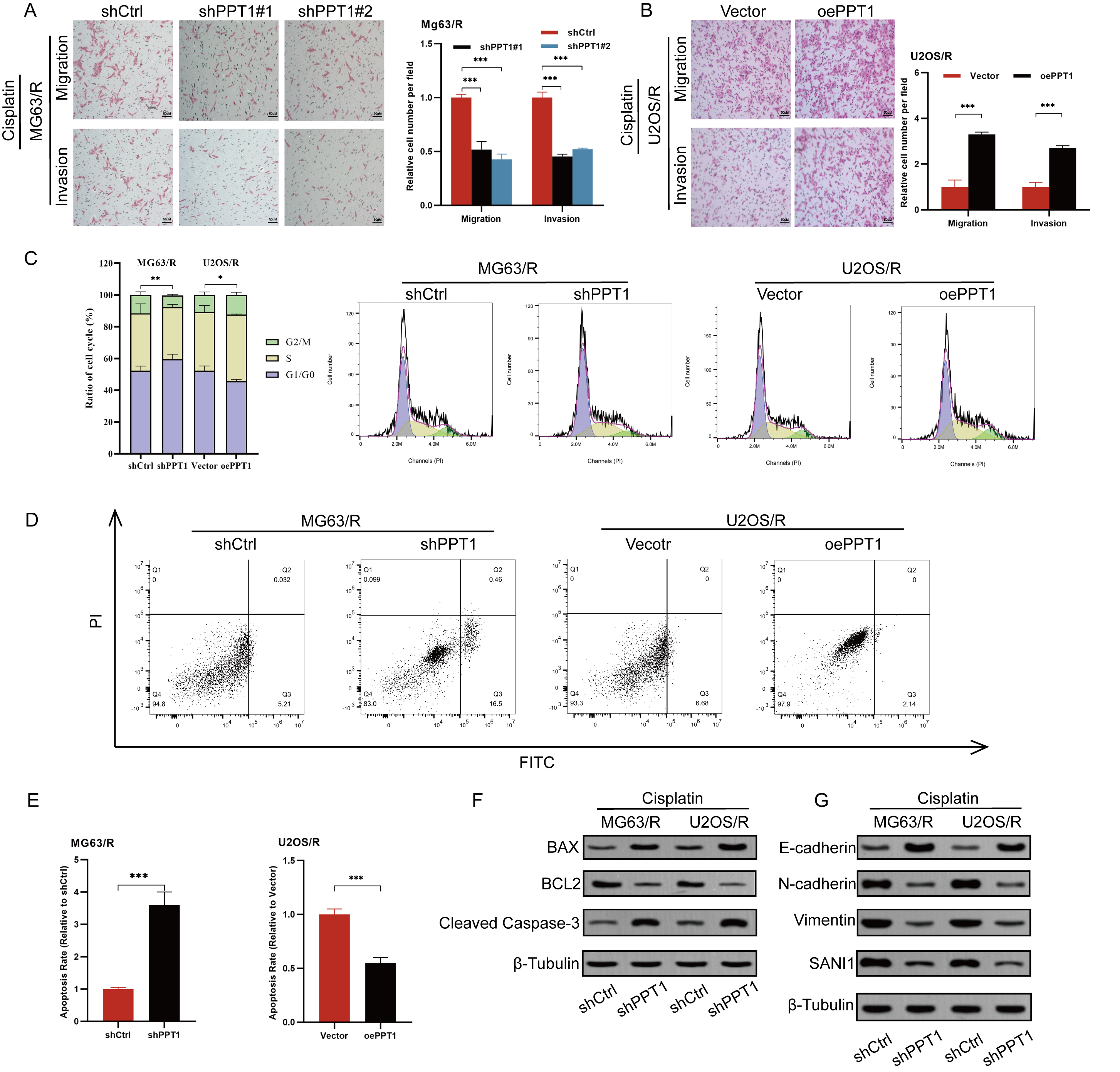

Supplement: Supplementary 1 — Figs. S1 to S9 Tables S1 to S3 [file research.0708.f1.zip › Figure S2.tif]

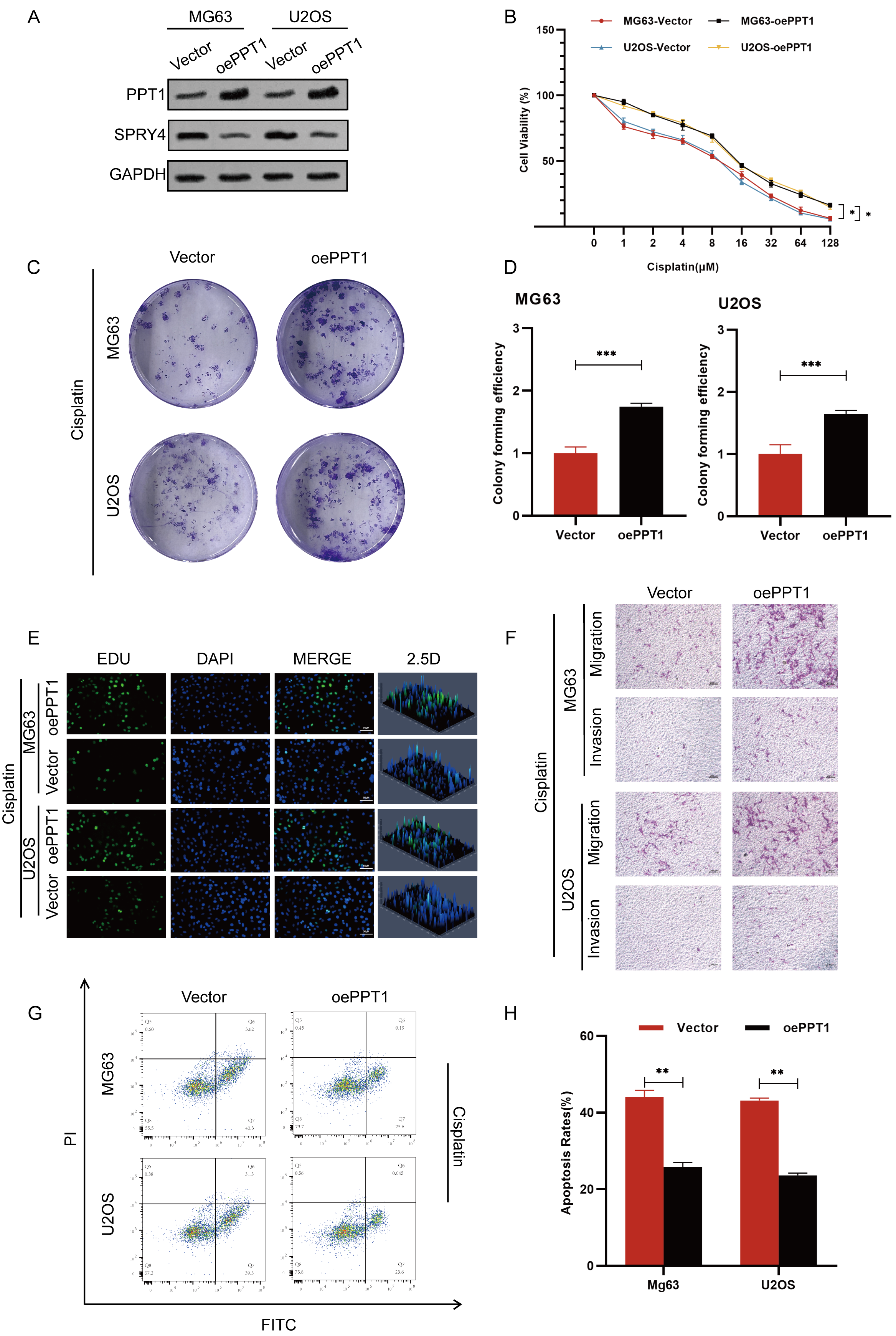

Supplement: Supplementary 1 — Figs. S1 to S9 Tables S1 to S3 [file research.0708.f1.zip › Figure S3.tif]

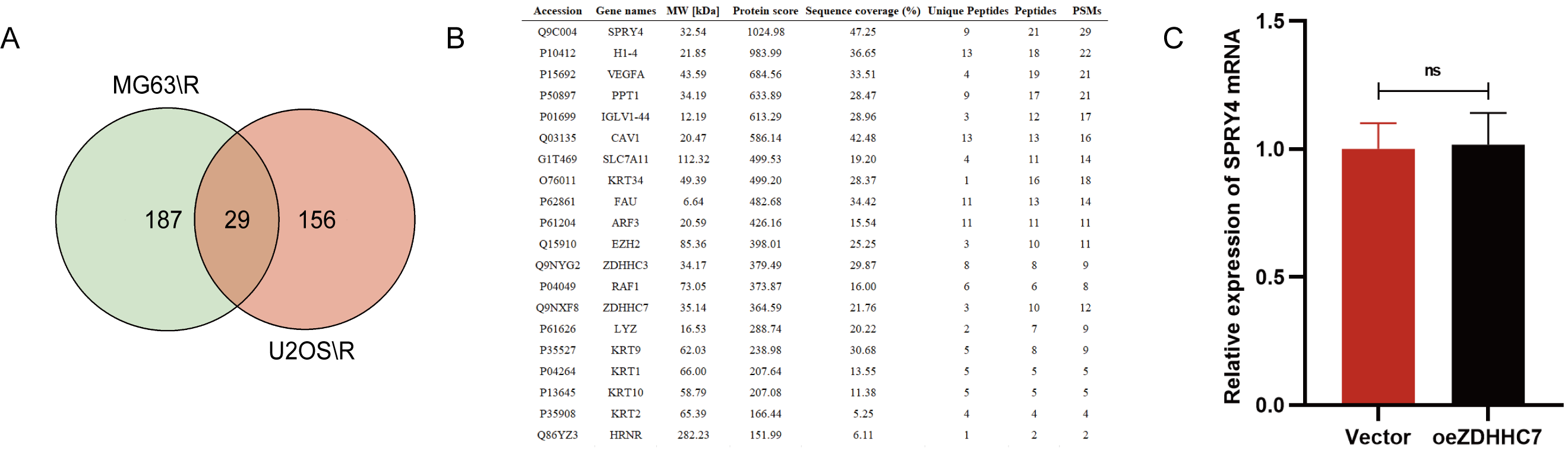

Supplement: Supplementary 1 — Figs. S1 to S9 Tables S1 to S3 [file research.0708.f1.zip › Figure S5.tif]

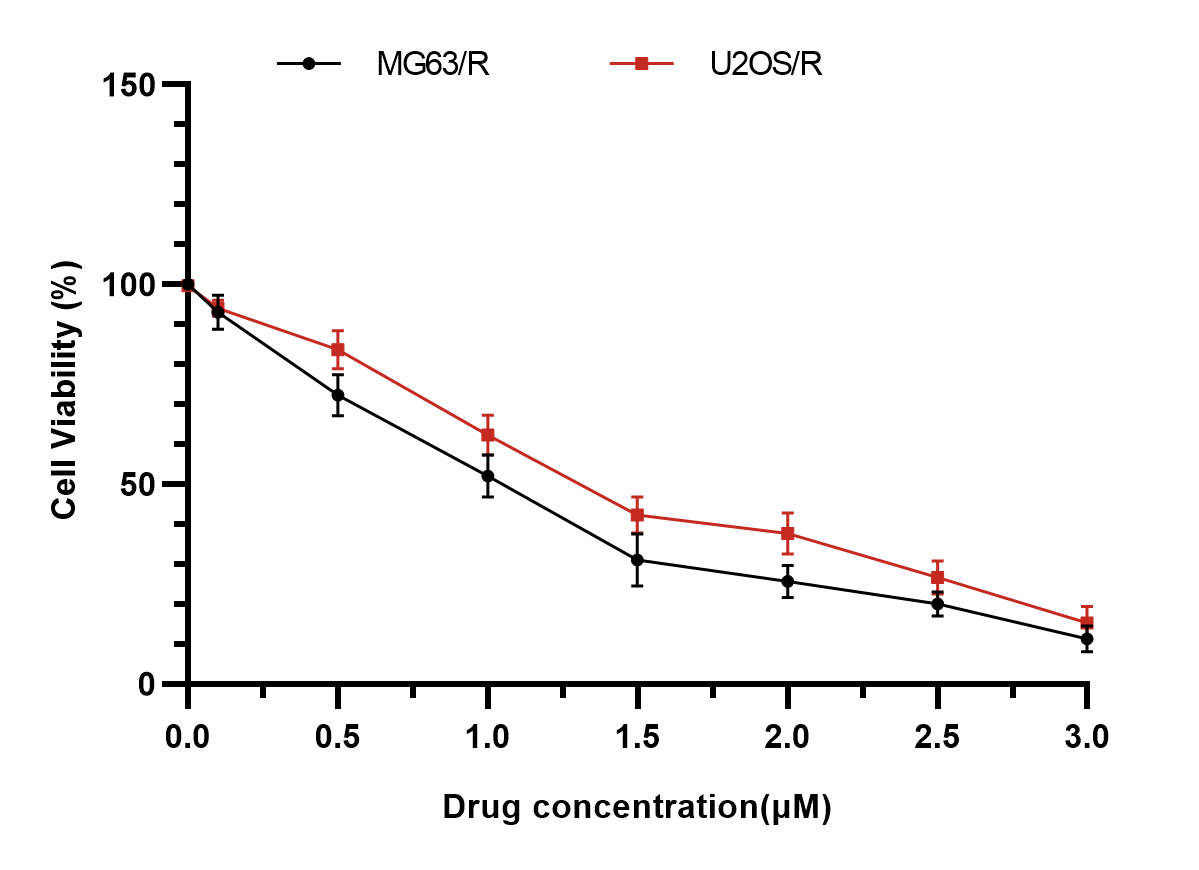

Supplement: Supplementary 1 — Figs. S1 to S9 Tables S1 to S3 [file research.0708.f1.zip › Figure S6.tif]

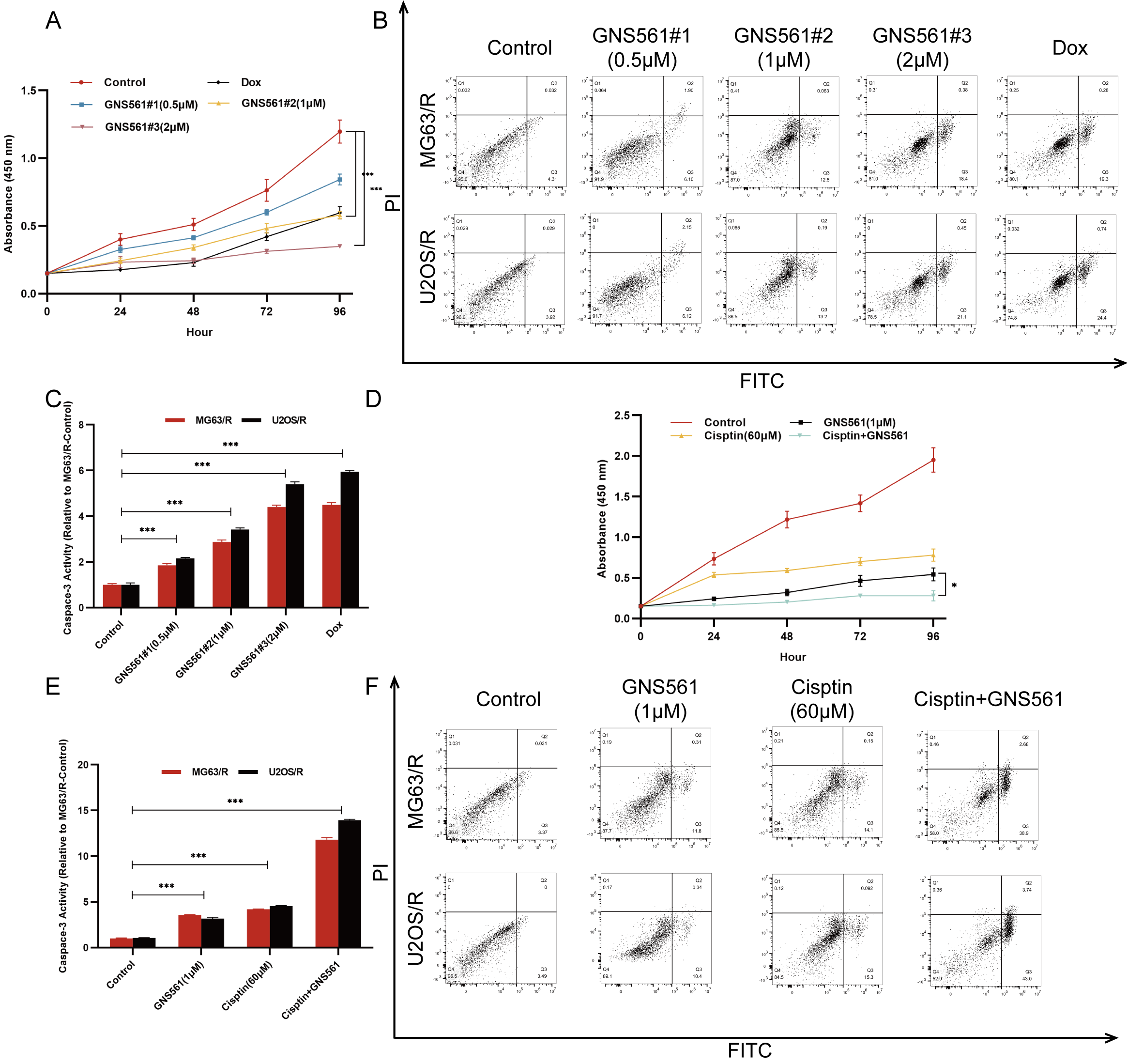

Supplement: Supplementary 1 — Figs. S1 to S9 Tables S1 to S3 [file research.0708.f1.zip › Figure S7.tif]

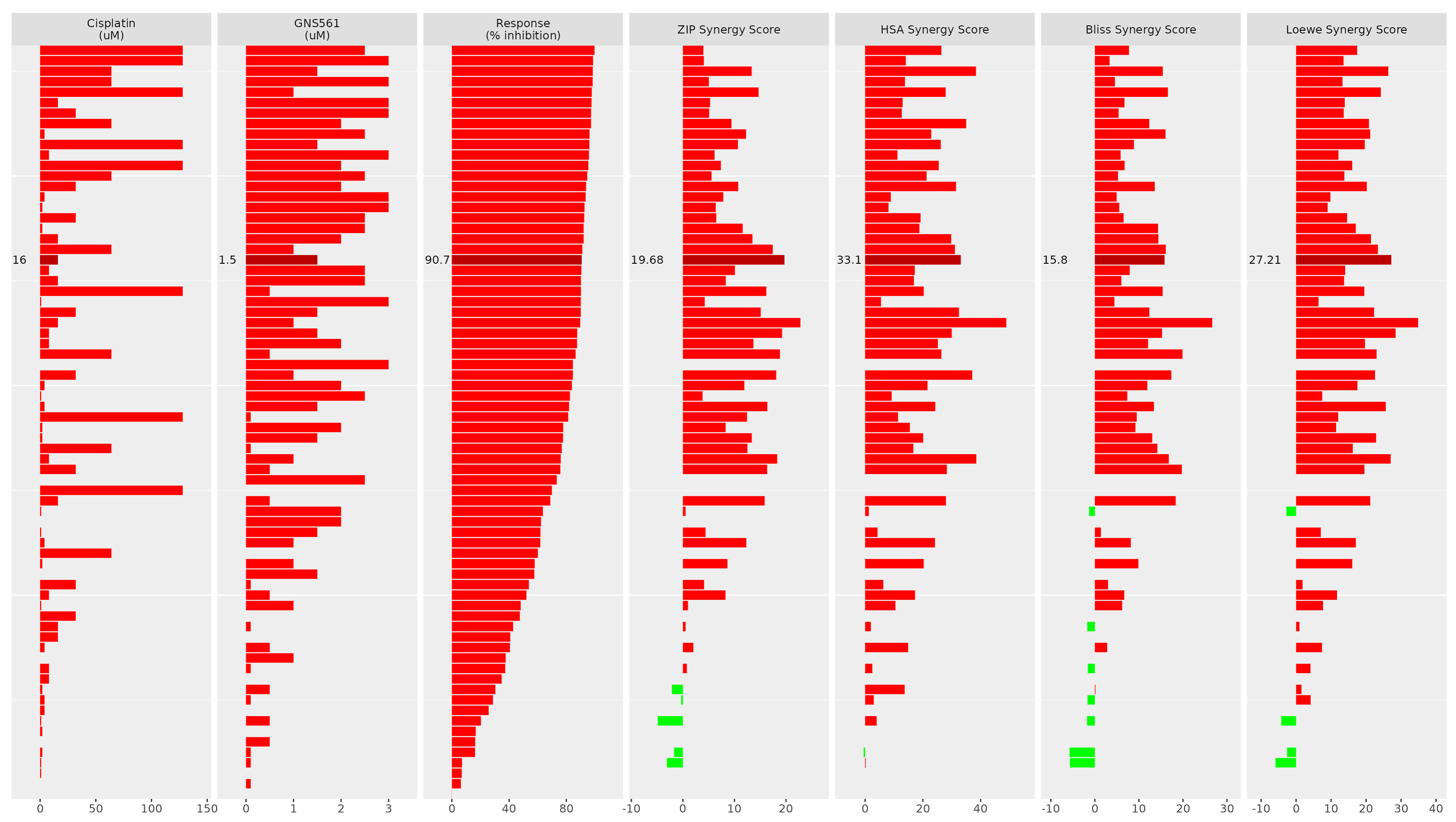

Supplement: Supplementary 1 — Figs. S1 to S9 Tables S1 to S3 [file research.0708.f1.zip › Figure S8.png]

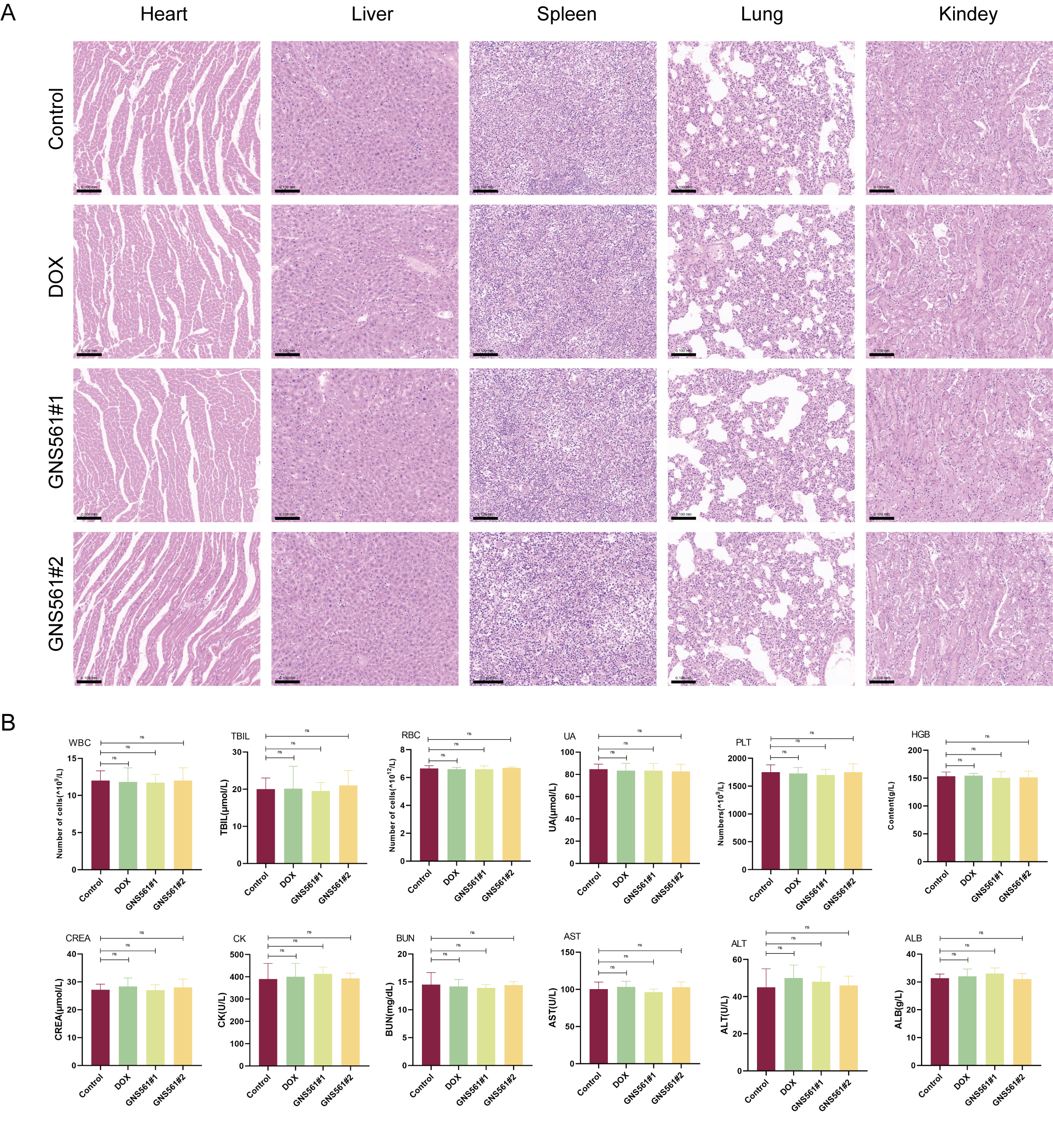

Supplement: Supplementary 1 — Figs. S1 to S9 Tables S1 to S3 [file research.0708.f1.zip › Figure S9.tif]
